# Multifunctional inorganic biomaterials: New weapons targeting osteosarcoma

**DOI:** 10.3389/fmolb.2022.1105540

**Published:** 2023-01-04

**Authors:** Dong Wang, Yi Peng, Yuezhan Li, Julius K. S. K. Kpegah, Shijie Chen

**Affiliations:** ^1^ Department of Spine Surgery, The Third Xiangya Hospital of Central South University, Changsha, Hunan, China; ^2^ College of Medicine, Nursing and Health Science, School of Medicine, Regenerative Medicine Institute (REMEDI), University of Galway, Galway, Ireland; ^3^ Xiangya School of Medicine, Central South University, Changsha, Hunan, China; ^4^ Shanghai Key Laboratory of Regulatory Biology, Institute of Biomedical Sciences and School of Life Sciences, East China Normal University, Shanghai, China

**Keywords:** osteosarcoma, material, black phosphorus, magnesium, zinc, copper, silver

## Abstract

Osteosarcoma is the malignant tumor with the highest incidence rate among primary bone tumors and with a high mortality rate. The anti-osteosarcoma materials are the cross field between material science and medicine, having a wide range of application prospects. Among them, biological materials, such as compounds from black phosphorous, magnesium, zinc, copper, silver, etc., becoming highly valued in the biological materials field as well as in orthopedics due to their good biocompatibility, similar mechanical properties with biological bones, good biodegradation effect, and active antibacterial and anti-tumor effects. This article gives a comprehensive review of the research progress of anti-osteosarcoma biomaterials.

## 1 Introduction

Osteosarcoma is the malignant tumor with the highest incidence among primary bone tumors (Mirabello et al., 2009; Rojas et al., 2021). It usually occurs in children and adolescents and often occurs in the distal femur, proximal tibia, and proximal humerus (Ritter and Bielack, 2010). For primary osteosarcoma without metastasis, the current clinical methods are surgery such as amputation, combined with radiotherapy and chemotherapy and others, with a high 5-year survival rate reaching 70% (Souhami, 1989; Heng et al., 2020). However, there are still 20% of patients who will have metastasis during treatment, especially prone to lung metastases (Ward et al., 1994; Meazza and Scanagatta, 2016). Once metastasis occurs, such as lung metastasis, the 5-year survival rate may be less than 30% (Tsuchiya et al., 2002). Until recently, extensive radical resection was used as the main treatment for osteosarcoma. More importantly, the continuous emergence of chemotherapy resistance in osteosarcoma further reduces the survival rate of patients, leading to low clinical benefits and poor postoperative quality of life for patients (Li et al., 2015). But, with the development of imaging, the application of angiography and interventional techniques, advances in neoadjuvant chemoradiotherapy and surgical techniques as well as rapid progress in immunotherapy, the treatment of osteosarcoma has undergone major changes and limb preservation surgery has an increasing ratio (Ferrari et al., 1997; Muscolo et al., 2005; Wong and Kumta, 2013; Takeuchi et al., 2019; Gill and Gorlick, 2021). Regrettably, to completely remove the tumor, a large number of tissues need to be removed during a limb-preserving surgery, which results in some challenges to the preservation and functional reconstruction of limbs. Therefore, the treatment of primary lesions is very important, especially those osteosarcomas that grow in the pelvis or the spine, which cannot be completely removed due to surgical limitations. Inevitably, there will be residual tumor tissue after surgery. However, the currently used autologous bone, allogeneic bone, and prosthesis only play the role of reconstruction, and cannot eliminate the local residual tumor tissue. Meanwhile, the various physical and chemical inactivation methods commonly used at present, such as neoadjuvant radiotherapy and chemotherapy, may damage the normal tissues while destroying the tumor tissue simultaneously, and these methods have no reconstruction effect. Therefore, finding an ideal material that can not only fill in but also kill the residual tumor cells, reducing the probability of recurrence, and metastasis and thereby promoting bone repair to treat osteosarcoma, has become a hot topic (Marques et al., 2014; Ma et al., 2020; Liao et al., 2021).

For this reason, many kinds of anti-osteosarcoma materials have emerged, and previous studies have mainly focused on the polymer compounds, such as poly (lactic acid-co-glycolic acid) (PLGA) and chitosan, etc., (Ma et al., 2014; He et al., 2022). These polymer compounds often require multiple modifications before they can function as anti-osteosarcoma, and they have no obvious advantages in promoting osteogenesis and mechanical properties (Table 1). For that matter, there’s a limitation to the clinical application of these polymers.

**TABLE 1:** The Comparison of features of different biomaterials.

Type of material	Anti-tumor effect	Toxicity	Inflammatory response	Bone regeneration capacity	Flaw
Magnesium	Phototherapy and nanoparticle targeting effects	Electrolyte disturbances	Less, depending on the concentration	Excellent	Rapid degradation rate and local hydrogen production
Zinc	nanoparticle targeting effects	Anemia and impaired immune function	Less, depending on the concentration	Good	Poor corrosion resistance
Copper	Photothermal therapy and nano drug delivery system	Liver function lesions and tubular necrosis and nephritis	Less, depending on the concentration	Good	Potential toxicity
Silver	nanoparticle targeting effects	Potential cytotoxicity	Less	General	Complex preparation process
Black phosphorus	Phototherapy and nanoparticle targeting effects	No obvious cytotoxicity	Less	Excellent	Unstable properties and low preparation efficiency
Poly (lactic acid-co-glycolic acid) (PLGA)	nanoparticle targeting effects	No obvious cytotoxicity	More	General	Poor mechanical properties

**FIGURE 1:**
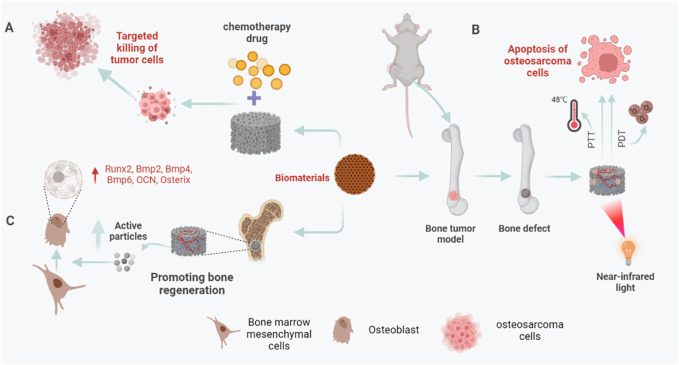
Biomaterials in bone reconstruction after osteosarcoma surgery. **(A)** Under near-infrared (NIR) light, biomaterials eliminate residual osteosarcoma cells through PTT and PDT to prevent a recurrence. **(B)** Biomaterials can be used as drug-loaded systems to target residual osteosarcoma cells, release chemotherapeutic drugs, and kill osteosarcoma cells. **(C)** After osteosarcoma resection, the 3D printed biomaterial scaffold can not only play the role of bone support and bone connection but also release some active particles, such as Ag^+^, Cu^+^, PO_4_^3-^, etc., to up-regulate the expression of osteogenesis-specific genes and promote bone regeneration.

But, in recent years, biological materials, such as black phosphorous (BP), magnesium (Mg), zinc (Zn), copper (Cu), silver (Ag), etc., have become more and more valuable in tissue engineering and orthopedics fields due to their good biocompatibility, mechanical properties similar to those of biological bones, biodegradation, antibacterial and anti-tumor effects (Figure 1) (Choi et al., 2018; Ambrosio et al., 2021). For instance, copper, magnesium and other metal ions can inhibit inflammation by promoting the polarization of macrophages from M1 to M2, which is conducive to bone regeneration and repair (Yang et al., 2021c; Diez-Tercero et al., 2021). Of these, the anti-tumor effect of biomaterials is mainly reflected in phototherapy. Phototherapy, as a minimally invasive and high-efficiency anticancer approach, has sparked extensive research interest (Hou et al., 2018). Phototherapy includes photodynamic therapy (PDT) and photothermal therapy (PTT) which have very different therapy mechanisms under the same stimulus. For PTT, a light at a specific wavelength irradiates photothermal agents, which heats up and kills tumor cells; however, in PDT, photosensitizers can produce large amounts of singlet oxygen (^1^O_2_) that can kill tumor cells under specific light exposure. Besides, several studies have found that local hyperthermia can activate heat shock proteins and promote the expression of osteogenesis-related genes, such as RUNX2, and BMP2, through the PI3K/AKT signaling pathway and ERK1/2 signaling pathway (Chen et al., 2015; Sayed et al., 2019; Wang et al., 2022). It can also increase the expression of alkaline phosphatase and promote bone differentiation (Shui and Scutt, 2001; Norgaard et al., 2006). These make phototherapy, especially PTT, attract the interest of many researchers in the process of bone repair after osteosarcoma surgery. At the same time, nanomaterials, such as nano-silver, BP nanosheets, etc., have shown great advantages in drug loading, tumor imaging, promoting osteogenesis, and anti-tumor properties (Gui et al., 2018; Qing et al., 2020; Ge et al., 2022; Zhu et al., 2022). Nanoparticles (NPs) usually refer to particles with a diameter between 1 nm and 100 nm, which can be used as biocatalysts, infrared absorbing materials, etc. Studies have shown that particles with a diameter of 5.5 nm–100 nm are not easily filtered by the kidney, but when passing through the tumor blood vessels, they can pass through the leaking blood vessels at the tumor, and then accumulate in large quantities in the tumor (Maeda, 2001; Poon et al., 2019). This is known as the enhanced permeability and retention (EPR) effect, which makes nanomaterials excellent for tumor therapy (Wu, 2021). Overall, this review describes recent advances and challenges in biomaterials for osteosarcoma treatment, inspiring future osteosarcoma research.

## 2 Magnesium, zinc, and their alloy

Mg, Zn, and their alloys exert excellent anti-tumor effects by influencing the metabolism and phenotype of tumor cells, inducing tumor cell proliferation inhibition, cell cycle arrest, and cell apoptosis (Krol et al., 2017; Zhou et al., 2021). Studies by Wu et al. have shown that magnesium-zinc alloy can inhibit the proliferation of osteosarcoma cell line U20S cells by arresting the G2/M phase of the cell cycle, and promote the apoptosis of U20S tumor cells through a mitochondrial-dependent pathway; at the same time, the alloy solution can inhibit the metastasis of U20S tumor cells through the MAPK pathway (Wu et al., 2016).

Moreover, Mg, as an essential trace element in the human body, indirectly affects mineral metabolism through its role in ATP metabolism and as a cofactor for more than 300 enzymes (Palacios, 2006). Therefore, Mg plays a more prominent role in bone tissue engineering by its excellent biocompatibility and biodegradability. Besides, the PTT of mg and the hydrogen generated from its degradation have also received increased attention in tumor therapy. On the ground, Long et al. have designed innovative multifunctional PLGA/Mg porous scaffolds with excellent biodegradability and biocompatibility by low-temperature three-dimensional (3D) printing technology (Long et al., 2021). *In vivo* experiments, Mg particles exhibit excellent photothermal effects for tumor eradication and Mg ions released from PLGA/Mg porous scaffolds could promote bone regeneration, which gives the PLGA/Mg scaffolds dual functions of inhibiting OS recurrence and continuously repairing bone defects. On the other hand, after intra-tumoral injection, Zhou et al. found that micro-scale Mg/PLGA exhibited stronger cytotoxicity, PTT, and anti-tumor effect than nano-scale Mg/PLGA (Zhou et al., 2021). This inspires the design of Mg scaffolds in the reconstruction of bone defects after osteosarcoma surgery. Besides, Zan et al. designed a magnesium-based biomaterial that can release hydrogen in a controlled manner, giving full play to the anti-tumor effect of Mg (Zan et al., 2022). Then, the generated hydrogen can promote the expression of tumor suppressor gene P53 and activate the mitochondria-related apoptosis pathway. At last, several studies also revealed that Mg can induce the apoptosis of osteosarcoma cells (MG63 and U2-OS cells) by shortening the half-life of Snail1 (Zan et al., 2020). However, although the human toxicity of Mg has been controversial, recent studies have reported that high concentrations of magnesium particles can inhibit osteoblast activity (Wang J. L. et al., 2020). This contradicts the potential osteogenic ability of Mg, which may be related to the concentration of Mg^2+^ in the bone microenvironment. The underlying mechanism is still unclear, and more research is needed to explore the metabolic mechanism of Mg^2^ in the bone microenvironment. In general, the toxicity study of magnesium-containing bone repair materials requires further studies (Table 1).

Nano-zinc biomaterials have also received increased attention in anti-tumor. For instance, He et al. (2018) revealed that zinc ions from ZnO NPs could suppress osteosarcoma cell proliferation by causing S phase arrest. Intercellular Zn ions also can target and damage the mitochondria, which could contribute to excessive reactive oxygen species (ROS) generation to promote apoptosis, which contributes to osteosarcoma cell death. They also found that there is an enhancing autophagosome formation and impaired lysosomal function with an upregulation of the LC3-II/LC3-I ratio after ZnO NPs treatment. Furthermore, there is crosstalk, in which apoptosis inhibition would contribute to autophagy, between apoptosis and autophagy in ZnO NPs-induced human osteosarcoma cell death. In addition, He et al. also revealed firstly an interplay between HIF-1α and the autophagy−Zn^2+^−reactive oxygen species (ROS)−autophagy cycle axis and confirmed that ZnO NPs could up-regulate HIF-1α in osteosarcoma cells mainly due to the combined effect of Zn^2+^ and ROS (He et al., 2020). Then, the studies *in vivo* experiments have shown that ZnO NPs could inhibit subcutaneous osteosarcoma proliferation with good biosafety by activating HIF-1α, apoptosis, and autophagy. Besides, Zn, like Mg, is mostly stored in the bones and may play a significant role in bone disease and osteogenesis (Palacios, 2006; Huang et al., 2020; Song et al., 2020). Based on this, a zinc-containing hydroxyapatite nanorod that promotes osteogenic differentiation of bone marrow mesenchymal cells in the absence of osteo-inductive factors is engineered (Fernandes et al., 2020). However, scaffolds of Mg, Zn, and their alloys often require a high-temperature fabrication process and are prone to corrosion after being placed in the body, which limits their clinical application (Table 1) (Koons et al., 2020). Therefore, while ensuring the degradability of metals, future research also needs to focus on the corrosion resistance of metallic materials. Additionally, to further exert the role of Zn and Mg biomaterials in bone repair after osteosarcoma surgery, the underlying molecular mechanisms need to be further explored.

## 3 Copper

As a constituent microelement of the human body and a cofactor for metalloenzymes, Cu plays an important role in human tissue regeneration, hemostasis, antibacterial, and anti-tumor (Harris, 1992; Palacios, 2006; Mendel et al., 2007; Yang J. et al., 2021). Thus, the imbalance of Cu in the internal environment will affect the normal function of tissues and organs, leading to adverse reactions such as anemia, malnutrition, neurodegenerative disease, and osteoporosis (Palacios, 2006; Brewer, 2010). Recently, some studies reported the design of scaffolds with antitumor and bone repair promotion by adding Cu elements (Kargozar et al., 2021; Solak et al., 2021). A new type of metal framework copper tetrakis (4-carboxyphenyl) porphyrin (Cu-TCPP) nanosheet interface structure is combined with β tricalcium phosphate (TCP) to make a Cu-TCPP-TCP scaffold (Dang et al., 2020). On one hand, the Cu-TCPP-TCP scaffold material uses near-infrared (NIR) irradiated light to exhibit photothermal performance, then killing the osteosarcoma cells by releasing heat energy. On the other hand, *in vitro* studies have found that the Cu-TCPP-TCP scaffold stimulates human bone marrow stromal stem cells (hBMSCs) and human umbilical vein endothelial cells (HUVEC), and significantly enhances the expression of osteogenic differentiation-related genes in hBMSCs and differentiation-related genes in vascular endothelial cells. In animal experiments, implanting a Cu-TCPP-TCP scaffold into a rabbit’s bone defect site can promote bone regeneration.

**FIGURE 2:**
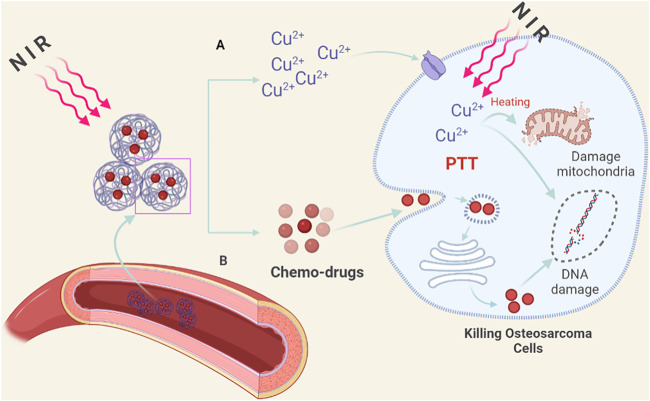
Nano-copper-based drug targeted delivery system. **(A)** Under the irradiation of NIR light, the nano-Cu-chemotherapy drug targeted delivery system decomposes and releases Cu^2+^ locally in the tumor, which can destroy the tumor cell membrane structure through PPT, kill tumor cells, and improve the sensitivity of chemotherapeutic drugs. **(B)** Under NIR light, the released chemotherapy drugs target tumor cells, causing DNA damage. At the same time, the damage to normal tissue cells is reduced.

In addition, it is worth noting that a nano-Cu-based drug-targeted delivery system will also bring new benefits to osteosarcoma patients (Figure 2). For example, Wang et al. (2016) reported that a smart therapeutic nanoplatform based on CuS@Zeolitic imidazolate framework-8 (ZIF-8) NPs have been developed (Gao et al., 2019). On this basis, they observe for the first time that after the loading of DOX the CuS@ZIF-8 NPs have synergistic chemo- and PTT effects on tumor cells *in vitro*/vivo studies. The low pH-sensitive property of the ZIF-8 framework makes a progress in integrating light/low pH triggered the release and chemo-photothermal therapy into one system which shows superior anticancer effects over the chem- or phototherapy alone. However, the toxicity of Cu limits further applications (Brewer, 2010; Ameh and Sayes, 2019). Several works of literature point out that Cu^2+^ can combine with a variety of organic substances and disrupt the normal homeostasis and physiological processes of the human body. In the body, Cu^2+^ is often accumulated in the liver, affecting liver metabolism and causing liver function lesions; in the kidney, it can cause tubular necrosis and nephritis (Table 1) (Chen et al., 2006). Based on this, more research is needed to explore the biodegradability or controlled release of Cu^2+^ in bone repair scaffolds.

## 4 Silver

Ag has been employed for biomedical purposes since ancient times owing to its anti-microbial properties (Xu et al., 2020). And, in the early 19th century, Ag preparations were developed for wound disinfection and burn care, and Ag nitrate was used for wound care and instrument disinfection. Regrettably, in the 1940s, the medical use of Ag gave way to the clinical use of antibiotics. But, with the development of nanotechnology, nano-silver particles (AgNPs) have received special interest due to their excellent antibacterial and antitumor effects. They are also used to promote wound repair and bone healing, as well as vaccine adjuvants, anti-diabetics and biosensors, etc., (Qing et al., 2018). Besides, several studies have also reported the role of Ag nanorods in photoacoustic (PA) imaging of inflammatory tissue, which is worthy of further exploration in the role of osteosarcoma imaging (Mei et al., 2020).

Then, AgNPs have been observed to exhibit good anticancer activities in breast cancer, cervical cancer, colon cancer, ovarian cancer, pancreatic ductal adenocarcinoma, lung cancer, hepatocellular carcinoma, melanoma, osteosarcoma, etc., (Chugh et al., 2018; Xu et al., 2020). And several studies have confirmed that the anticancer activity of AgNPs varies in various sizes, shapes, and doses/concentrations in different cancer cells (Jo et al., 2015; Dziedzic et al., 2016). In general, AgNPs show broad-spectrum anticancer effects through size, dose/concentration, and time-dependent ways. Smaller AgNPs can induce enhanced endocytosis and more significant cytotoxicity and genotoxicity. Compared with other shapes, spherical AgNPs exhibit greater cytotoxicity due to a higher surface-volume ratio. And higher doses of AgNPs generally lead to more apoptosis than lower doses. On the basis, taking advantage of the lack of function of the P53 gene in a variety of tumors, Kovacs et al. proposed a clever idea based on a therapeutic strategy that stimulates the function of P53, prepared Ag nanoparticles, and tested their cytotoxic effect on the osteosarcoma cells (U20S, Saos-2) which lack the function of P53 tumor suppressor gene (Kovacs et al., 2016). The results showed that the mitochondrial structure and function of osteosarcoma cells treated with citric acid-coated AgNPs were disordered, and the apoptosis rate was increased, indicating that the NPs did not depend on the functional state of P53 in killing the osteosarcoma cells. This feature makes AgNPs become another choice for chemotherapy strategies.

Furthermore, Recent research further explores the use of smaller-scale angstrom silver (one-tenth of a nanometer, AgAP) in cancer therapy. On the one hand, Xie et al. announced for the first time the broad-spectrum anti-cancer properties of AgAP and the body’s good tolerance (no obvious side effects) to AgAP (Wang Z. X. et al., 2019). It is precise because smaller particles have greater cellular toxicity, Xie et al. speculated that AgAP particles have stronger anti-tumor effects (Jo et al., 2015). And they independently developed automatic “metal vapor-condensation” equipment based on physical high temperature and pressure gasification methods for the preparation of angstrom material. At the same time, by using fructose to modify AgAP, AgAP stably existing in the solution was obtained. The results of cell and animal experiments show that AgAP injection exhibits killing effects on lung cancer, pancreatic cancer, and other tumors, but has no obvious toxic or side effects on normal tissues. Furthermore, Xie et al. found that fructose-coated angstrom silver (F-AgÅPs; 9.38 nm ± 4.11 nm) can effectively kill a variety of osteosarcoma cell lines and primary osteosarcoma cells (Hu et al., 2020). Compared with cisplatin, one of the first-line drugs for osteosarcoma treatment, F-AgÅPs can more effectively inhibit the growth of osteosarcoma transplanted subcutaneously in nude mice and *situ* osteosarcoma, reduce the damage of *in situ* osteosarcoma to bone and inhibit its metastasis to lung, and has no obvious effect on normal cells and tissues at therapeutic doses. Tissue distribution and metabolism results show that after intravenous injection of F-AgÅPs, Ag presents a high level of accumulation in tumor tissues and is mainly excreted through feces (the excretion rate through feces after one week is about 68% of the injected dose). On the other hand, tumor cells still mainly use glycolysis rather than mitochondrial oxidative phosphorylation to break down glucose and produce ATP (Warburg effect) even under the condition of adequate oxygen supply (Bonnet et al., 2007). Aerobic glycolysis can prevent tumor cells with active oxidative metabolism from producing excessive ROS and protect them from apoptosis caused by ROS (Stacpoole, 2017; Woolbright et al., 2019). Pyruvate dehydrogenase kinase (PDK) is a mitochondrial enzyme that can selectively phosphorylate pyruvate dehydrogenase (PDH) E1α subunit to inactivate it, thereby prompting the cell glucose metabolism to switch from aerobic oxidation to glycolysis. Mechanism studies have shown that F-AgÅPs can activate PDH by inhibiting PDK so that the glucose metabolism state of osteosarcoma cells changes from glycolysis to mitochondrial aerobic oxidation, thereby selectively inducing osteosarcoma cells (rather than normal cells) to generate ROS-mediated apoptosis (Hu et al., 2020).

Moreover, several studies have reported on the creation of nanocomposites that promote bone regeneration (Xu et al., 2020). AgNPs are one of them, exhibiting an excellent ability to promote bone repair. For example, Zhang et al. have uncovered that AgNPs induce proliferation and osteogenic differentiation of MSCs *in vitro*, stimulate callus formation and accelerate the healing of fractured bone in an osteogenic mouse model (C57BL/6 mice) (Zhang R. et al., 2015). Mahmood et al. have confirmed that AgNPs significantly enhanced osteocyte mineralization and differentiation in MC3T3-E1 cells (an *in vitro* model) compared with several other NPs and many genes related to the osteogenesis pathway were expressed in both control cell cultures and those exposed to AgNPs (Qing et al., 2018). However, in response to AgNPs exposure, there was a significant increase in key factors including Bmp4, Bmp6, and Fosl1, associated with osteoclast pathways. At last, they revealed that AgNPs accelerated the differentiation and proliferation of McT3-e1 cells by the differential expression genes (DEGs) and functional analysis. Besides, Han et al. fabricated AgNPs-loaded Gel hydrogels (AgNPs/Gel) by a simple method under sunlight using gelatin as a stabilizing agent which shows an excellent effect on bone regeneration and fracture treatment (Han et al., 2021). These innovative explorations reveal the advantages of AgNP as a multifunctional biomaterial, which may help to solve the problem of large bone defects and recurrence after osteosarcoma surgery.

In addition, Xie et al. also first reported that Carbomer gel loaded with AgAPs (simplified as AgAPs gel) can promote the repair and regeneration of damaged skin by potent sterilization and reducing inflammation (Chen et al., 2020). It has been proved that AgAPs gel can effectively kill a variety of bacteria *in vitro* (including *Pseudomonas aeruginosa*, methicillin-resistant, and methicillin-sensitive *Staphylococcus aureus*), inhibit the bacteria colonization in skin defect sites of diabetic mice, and the large scalded area of the common mice, reduce the inflammation of the wound, and thus accelerate the healing of the wound. The AgAPs r gel of therapeutic dose has no significant effect on the *in vitro* activity of normal skin repair-related cells and the multiple physiological functions and organ tissue structures in mice and the topical application of AgAPs gel for several days did not cause Ag accumulation in other organs in mice. This significant discovery will inspire us to further explore the potential and more meaningful applications, such as bone repair, of AgAPs s in nanomedicine.

However, despite the remarkable effects of AgAPs and AgNPs in anti-inflammatory, anti-tumor, and promoting bone repair, the process requirements and high energy consumption of its fabrication also hinder wide clinical applications (Zhang X. F. et al., 2016). Moreover, due to the natural high affinity of Ag to sulfur, AgAPs and AgNPs can bind proteins or sulfur-containing macromolecules *in vivo*, thereby promoting membrane damage, ROS generation, protein oxidation, and denaturation, mitochondrial dysfunction, DNA damage and inhibition of cell proliferation (Table 1) (Tortella et al., 2020). But, there is no doubt that AgNPs biomaterials still hold great promise in bone tissue engineering, and further exploration to obtain slow-release or locally degraded silver biomaterials may help to address these challenges.

## 5 Black phosphorus

**FIGURE 3:**
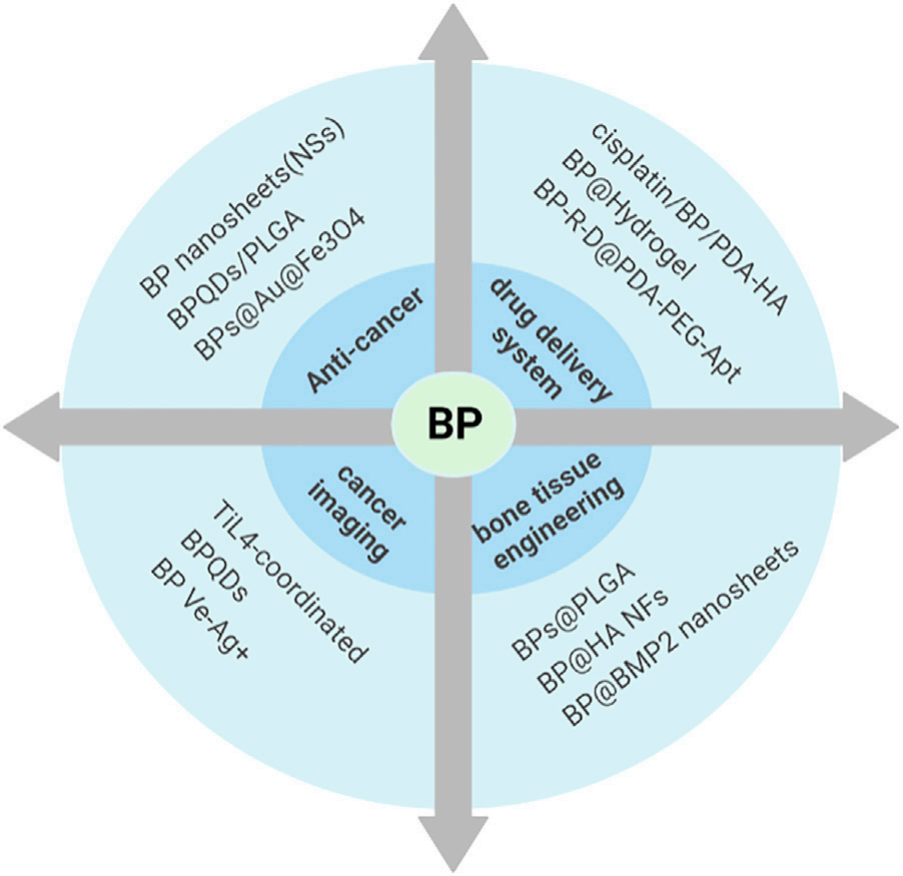
Biomedical applications of BP. With its unique structure, BP has been more and more favored in anti-tumor, drug-targeted delivery, photoacoustic imaging, and bone tissue engineering after various modifications.

**FIGURE 4:**
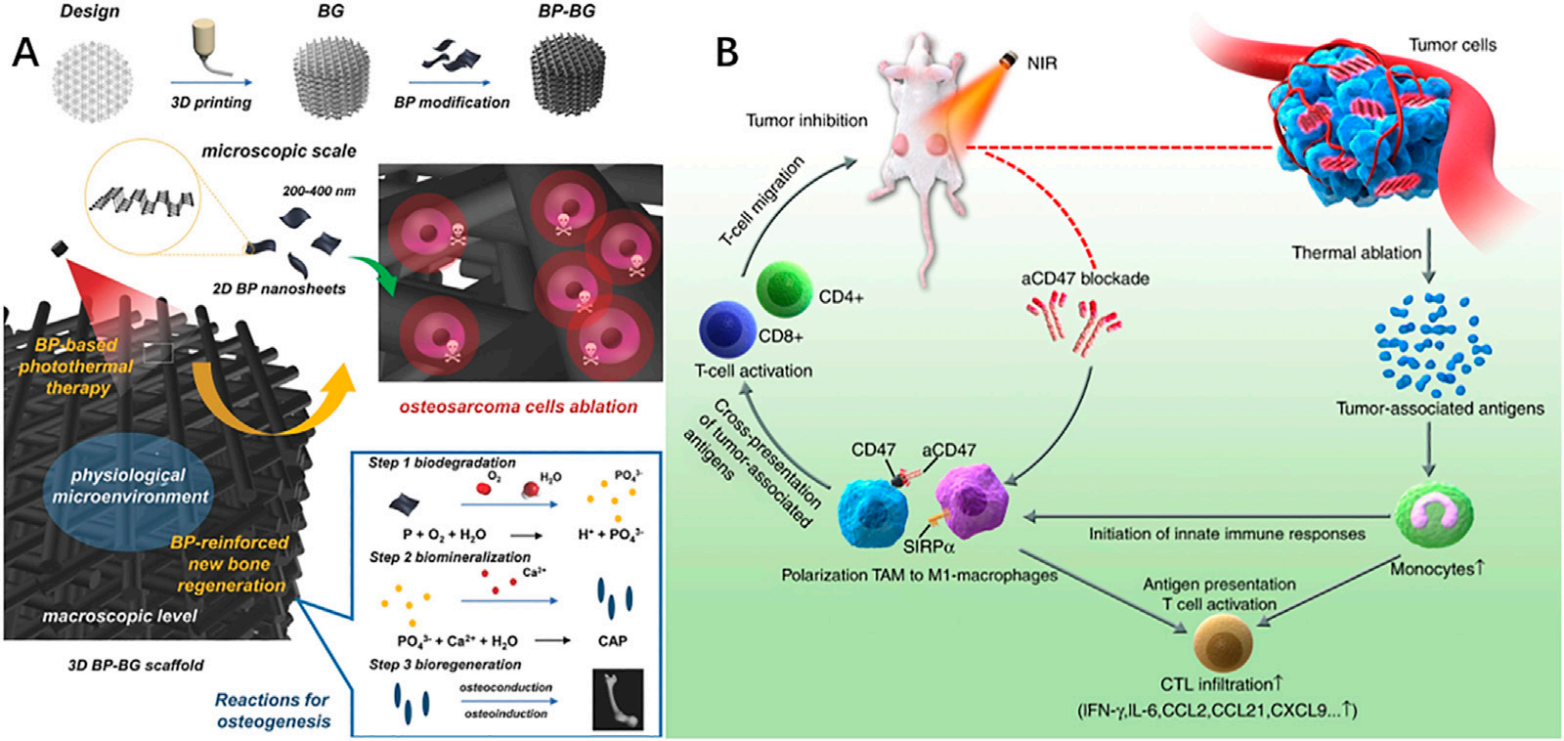
**(A)** 2D-Black-Phosphorus-Reinforced 3D-Printed Scaffolds. Schematic illustration of the fabrication process for BP-BG scaffold and the stepwise therapeutic strategy for the elimination of osteosarcoma followed by osteogenesis by BP-BG. **(B)** Black phosphorus-based photothermal therapy with aCD47-mediated immunotherapy. Black phosphorus in combination with anti-CD47 antibody activates innate and adaptive immunity and promotes local and systemic anticancer immune responses, thereby providing a synergistic enhancement in inhibiting tumor progression and suppressing metastatic cancer. **(A)**: Yang et al. (2018). 2D-Black-Phosphorus-Reinforced 3D-Printed Scaffolds: A Stepwise Countermeasure for Osteosarcoma. *Adv Mater 30(10)*. doi: 10.1002/adma.201705611. **(B)**: Xie et al. (2020). Black phosphorus-based photothermal therapy with aCD47-mediated immune checkpoint blockade for enhanced cancer immunotherapy. *Light Sci App*l 9, 161. doi: 10.1038/s41377-020-00388-3.

BP nanomaterials, also known as phosphenes, a new member in the two-dimensional (2D) material family, have sparked considerable research interest (Kou et al., 2015). In the monolayer BP, each phosphorus atom is covalently linked with three adjacent phosphorus atoms to form a puckered phosphorus layer structure, and the phosphorus layer and surface are closely bonded by the van der Waals force (Choi et al., 2018). Compared with other 2D nanomaterials, the BP at the nanometer level has a fold structure and a bilayer structure along the Zigzag direction, which makes the BP have a higher specific surface area. This structural anisotropy contributes to its excellent properties, including its optical properties, mechanical properties, electrical conductivity, thermoelectric properties, and properties that distinguish its topology from other 2D materials. In addition, another zero-dimensional structure nanomaterial of BP, black phosphorus quantum dots (BPQDs), was successfully synthesized by chemical methods and attracted wide attention (Sun et al., 2015). In 2015, Zhang et al. achieved the first breakthrough in the preparation of BPQDs (Zhang X. et al., 2015). Using a facile liquid-phase sonication technique to fabricate BPQDs, Zhang and colleagues successfully prepared BPQDs with uniform size and better dispersion. Then, BPQDs have exhibited significant application in biomedicine (Gui et al., 2018). In general, BP nanomaterials have attracted widespread attention for biomedical applications, such as PTT, PDT, drug delivery, bioimaging, and tissue engineering since it was first discovered in 2014 (Figure 3). For example, Yang et al. (2018) based on the PTT and osteogenesis of BP, made a breakthrough in integrating 2D BP nanosheets into 3D printed bioglass (BG) scaffolds (Figure 4A). From the micro-scale to the macro-level, Yang successfully prepared multifunctional biomaterials with osteogenic and anti-osteosarcoma properties *in vitro* and *in vivo*. And, in this section, we will discuss the biomedical applications of the properties of black phosphorus.

### 5.1 Anticancer properties of BP

Owing to its excellent photothermal conversion properties, black phosphorus has been explored and used as a PTT agent or a photosensitizer in PDT *in vivo* cancer therapy (Qi et al., 2021).

Shao et al. (2016) used the emulsification solvent volatilization method to prepare core-shell structured nanospheres with high polymer (PLGA) encapsulating BPQDs. PLGA is a degradable hydrophobic biomedical polymer, and the formed polymer shell can isolate the internal BPQDs from the physiological environment, ensuring the stable performance of the BPQDs during the treatment process. After the PTT is over, the BPQDs will be slowly released and degraded with the gradual degradation of the PLGA shell, and then safely metabolized out of the body. Cell and animal experiments show that BPQDs/PLGA has good biological safety and passive tumor targeting, and shows high PTT efficiency. Five minutes of near-infrared light can effectively kill tumors. This promotes the actual clinical application of PTT. In addition, several reports have revealed that a large number of tumor antigens and alarmins, acting as an endogenous stimulatory signal that can improve tumor immunogenicity, are produced when BP kills tumors through PDT (Li W. et al., 2019; Alzeibak et al., 2021).

More importantly, recent studies have attempted to combine BP phototherapy with tumor immunotherapy, to achieve innovative breakthroughs in the treatment of osteosarcoma. Generally speaking, in the tumor microenvironment, when interacting with signal regulatory protein-alpha (SIRPα) which is expressed on macrophages, CD47 can realize the function of “do not eat me” (Liu M. et al., 2019). On the ground, Xie et al. (2020) found that BP-based PTT plus in combination with anti-CD47 antibodies (aCD47) can prompt the repolarization of tumor-associated macrophages (TAMs) from M2-like to M1-like macrophages, block the “do not eat me” signal of CD47-SIRPα in tumor cells and promote phagocytosis of macrophages (Figure 4B). Then, activated macrophages may enhance the local cross-presentation of tumor-specific antigens and facilitate the production of tumor antigen-specific T cells against distant metastatic tumor cells. Yang et al. (2017) have fabricated a novel nanocomposite, showing highly biocompatible and excellent tumor suppression due to synergistic PTT and PDT mediated by low-power near-infrared lasers, by assembling iron oxide (Fe_3_O_4_) NPs and Au nanoparticles on BP sheets (BPs@Au@ Fe_3_O_4_). Besides, there are also several reports that more precise and efficient PTT and PDT have been obtained by modifying black phosphorus or combining it with other materials. For example, by combining the plasmonic photothermal effect of Au nanoparticles with MRI of Fe_3_O_4_ NPs for the first time, BPs@Au@ Fe_3_O_4_ shows a more significant photothermal treatment effect and more selective targeted therapy. However, although BP-based phototherapy has achieved some gains in cancer treatment, its clinical application in osteosarcoma remains a formidable challenge, and more research is needed to achieve this translation. Meanwhile, the damage of PTT of BP to the normal tissue around the tumor has also sparked controversy, and the subsequent mild photothermal therapy may be its potential solution (Jiang et al., 2021).

### 5.2 BP-based drug delivery system

For cancer, traditional drug therapy often has more or fewer defects, such as easy degradation, adverse reactions, and lack of targeting ability. But, Over the past decade, as 2D nanomaterials, such as graphene oxide (GO), BP, and molybdenum disulfide with various unique physical and chemical properties have been widely studied, more and more research is turning interest in these biomaterials to overcoming these challenges (Biju, 2014; Yin et al., 2017; Liu et al., 2018). Among them, BP has also been widely discussed as a drug delivery system with a large surface area, fold-like structure, good biodegradability, and active nano-interactions (Tao et al., 2017; Wang S. et al., 2019; Liu W. et al., 2021).

On the one hand, the current exploration is mainly to modify BP through polymer compounds, such as hydrogels, and PLGA, to increase the anti-tumor drug carrying capacity of BP nanosheets, increase the stability of the BP structure, and achieve a controllable and sustained drug release (Tao et al., 2017; Choi et al., 2018). Qiu et al. (2018) used the non-contact probe ultrasonic liquid peeling method to successfully prepare two-dimensional layered phosphorene nanosheets, and integrate them with anticancer drugs into the biodegradable temperature-sensitive hydrogel material to prepare black phosphorus hydrogel material. Under the irradiation of near-infrared light, the black phosphorus in the material can generate local high heat, which can not only kill tumor cells directly through photothermal action but also target tumor tissue to release drugs. The rate of drug release can be more precisely controlled by various parameters such as the intensity of the laser light field, irradiation time, and black phosphorus concentration, and ultimately achieve the effect of treating tumors. Besides, Li Y. et al., 2021 have designed a BP nanosheet-based nano-assembly containing cisplatin and used polydopamine (PDA) and hyaluronic acid (HA) to modify the surface of black phosphorus, achieving higher stability, a stronger photothermal effect, and targeting ability. Then in the tumor microenvironment, cisplatin/BP/PDA-HA (CBPH) would start to degrade and release cisplatin in a controlled manner by responding to internal or external stimuli, such as low pH, hydrogen peroxide, and near-infrared light. Therefore, *in vivo* experiments further revealed that there is a greater accumulation of cisplatin in tumor tissue and smaller primary tumors, and fewer lung metastases under the stimulation of light.

On the other hand, it has been pointed out that after black phosphorus is taken up by tumor cells, the active phosphorus produced can play an anti-tumor effect. Studies by Geng et al. (2020) have found that due to the vigorous endocytosis of cancer cells compared to normal cells, faster metabolic rate, and strong oxidative pressure, BP nanosheets are easily taken up by cancer cells rather than normal cells through endocytosis, and are rapidly degraded, resulting in the production of a lot of phosphate ions in the cell. This process leads to changes in the internal environment of cancer cells, causing G2/M phase blockade, thereby effectively inducing apoptosis and autophagy in cancer cells, which brings a better therapeutic effect than the traditional chemotherapy drug doxorubicin (DOX) *in vitro* and *in vivo* experiments. The research team named this selective killing of cancer cells derived from the natural biological activity of black phosphorus, “Bioactive Phosphorus-based Therapy” (“BPT”).

So, the deepening of biomaterial research also provides new therapy options for the treatment of recurrent or metastatic osteosarcoma.

### 5.3 BP for cancer imaging

Recently, with high image contrast and sensitivity, high spatial resolution with depth up to several centimeters, and depth resolution 3D imaging, photoacoustic (PA) imaging has attracted widespread interest, then reducing unnecessary biopsies and facilitating image-guided therapy (Lemaster and Jokerst, 2017). However, although PA imaging has been researched to be superior to many other traditional optical imaging techniques, there are still many problems to be solved in its clinical application. In the early stage of the tumor, the PA signal from the tumor is very low, so we need a contrast agent to enhance the signal and obtain more accurate *in vivo* imaging of PA. Lately, several studies have reported nanomaterials, such as metals, semiconductors, and reduced graphene oxide (RGO) with NIR absorption as contrast agents for better imaging, but have been limited by their potential toxicity in clinical applications (Fu et al., 2019).

Fortunately, BP is emerging as an alternative material for contrast agents in photoacoustic imaging, considering its excellent electronic and optical properties (Gui et al., 2018). On the other hand, the degradation of black phosphorus to phosphate *in vivo* avoids potential toxicity limitations. In the way that mixes BPQDs prepared by a liquid exfoliation technique and titanium ligand (TiL_4_) in N-methyl-2-pyrrolidone (NMP) at room temperature for 15 h, Sun et al. (2017) have fabricated TiL4-coordinated BPQDs, showing better PA performance *in vivo*. Titanium ligand sulfonates made BPQDs more stable in aqueous media through the surface ligands of BPQDs. In addition, a few studies have further reported that BPQDs exhibit higher spatial resolution, deeper penetration, lower optical absorption and scattering from biological tissues, and lower autofluorescence for PA imaging in the second NIR (NIR-Ⅱ, 950 nm–2,000 nm) window (Fu et al., 2019; Xu et al., 2019). On this basis, an exogenous NIR stimulus responsive BPQDs vesicle (BP Ve) was constructed by Li Z. et al. (2020) and can chelate and release Ag^+^ ions. Then Ag^+^ ions-coupled BP Ve shows not only more effective NIR-II PA imaging ability but also synergistic photodynamic/Ag^+^ therapy owing to enhanced light absorption and PA intensity in the NIR-II window. Therefore, more research is needed to further explore the potential value of BP in oncology, to address these challenges in the early diagnosis and treatment of recurrent or metastatic osteosarcoma.

### 5.4 BP for bone tissue engineering

**FIGURE 5:**
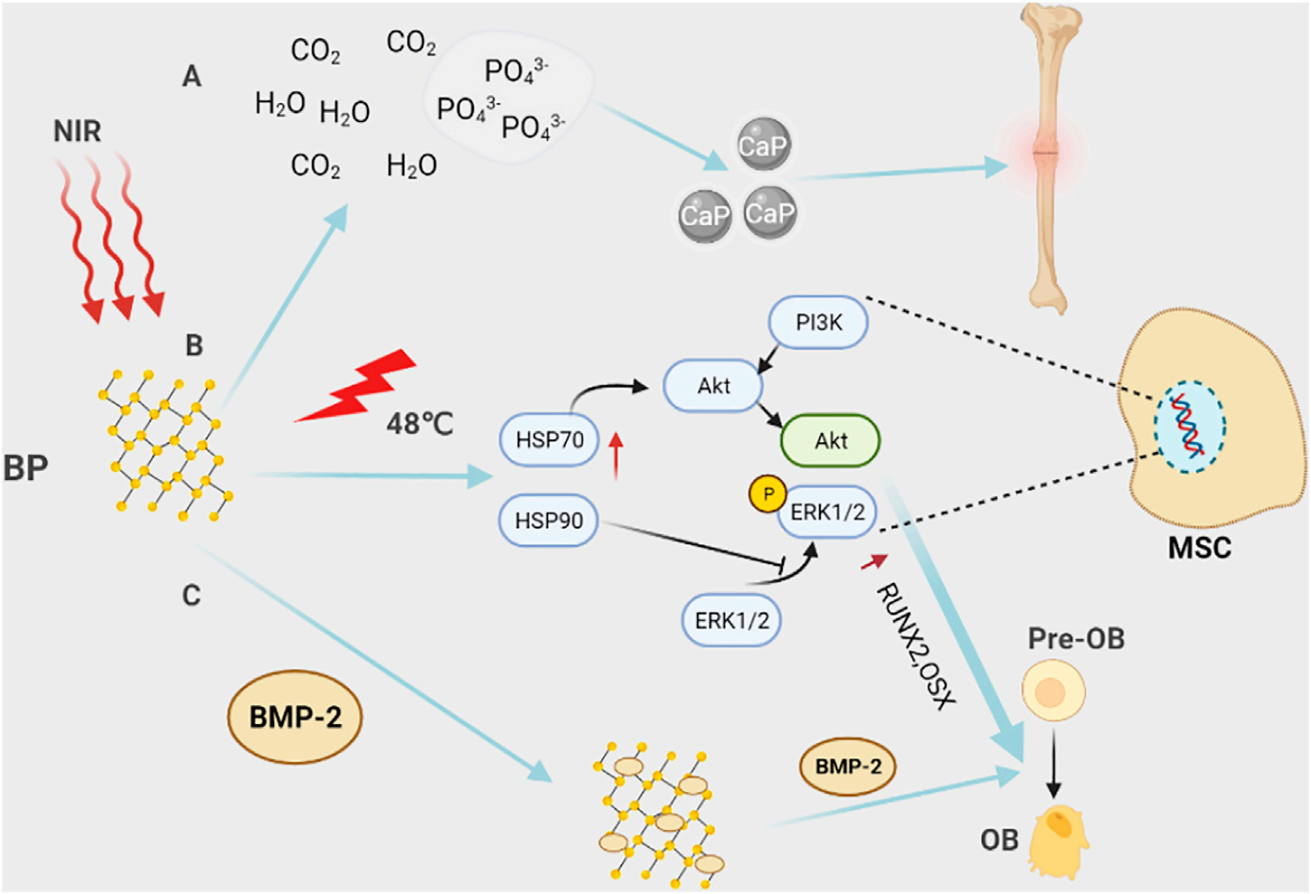
Black phosphorus in bone tissue engineering. **(A)** Black phosphorus is completely degraded in the body into harmless H_2_0, CO_2_, and PO_4_^3-^. PO_4_^3-^ is one of the basic components of the bone matrix, which is beneficial to promote bone repair. **(B)** Under NIR light, BP locally generates heat through the photothermal effect, activates heat shock proteins, and then promotes the expression of osteogenic-specific genes, such as RUNX2, BMP2, and the differentiation of pre-osteoblasts into osteoblasts through activating ERK1/2 signaling pathway and PI3K/AKT signaling pathway. **(C)** With their unique structure, black phosphorus nanosheets can carry BMP2, etc., and regulate bone formation in the bone microenvironment. For example, BMP2 promotes the differentiation of pre-osteoblasts into osteoblasts.

According to recent research, BP nanomaterials have many advantages in bone regeneration. First, compared with other 2D materials, they have good biocompatibility and are biodegradable in the physiological environment (Qing et al., 2020). The BP is completely biodegradable, and the final degradation products are harmless H_2_O, CO_2_, and PO_4_^3-^, which can be used as essential bone components (Tong et al., 2019). Based on this, the preparation of a photo-responsive BP@Hydrogel provides an *in situ* mineralized model controlled by timing and direction of light, exhibiting high potential for mechanical properties and bone induction (Shao et al., 2020). Therefore, this platform provides a good mimicking extracellular matrix (ECM) microenvironment for promoting osteoblast differentiation and bone regeneration (Figure 5A).

Besides, due to the strong light absorption capacity of BP in the NIR, BP based nanomaterials or 3D printed scaffolds have a stable and reliable light-controlled release mode, to achieve the purpose of targeted and sustained release. Simultaneously, its unique photothermal conversion ability can both promote bone regeneration and repair by up-regulating alkaline phosphatase (ALP) and heat shock proteins (HSP) through hyperthermia, and also killing tumor cells by increasing local temperature (Figure 5B) (Qing et al., 2020). In this regard, a chitosan/hydroxypropyl trimethylammonium chloride chitosan/hydroxyapatite/black phosphorus (CS/HC/HA/BP) composite scaffold is designed to take advantage of these characteristics of black phosphorus, aiming to deal with the clinical problems of tumor recurrence, bone defect and chronic bone loss after bone tumor surgery (Zhao et al., 2023).

At last, due to their large surface area and fold-like structure, BP nanomaterials have greater active agent-carrying capacity including various drugs, biomolecules, and nanoparticles. For the first time, Li Z. et al. (2021) designed a Ca^+^ ion-supplying BP-based 3D nanocomposite fiber scaffold *via* microfluidic technology, which brings new therapeutic prospects to elderly patients with bone defects or bone damage caused by calcium loss. In addition, by integrating BP nanosheets and hydroxyapatite-silica (SiO_2_) nanoparticles onto 3D PLGA nanofibers, the scaffold has a better Ca/P ratio, variable pore size distribution, and highly porous interconnected structure, providing a better microenvironment for the bone repair compared to other composites based on BP. Further, based on black phosphorus can provide a negative surface and strong bone morphogenetic protein_2_ (BMP_2_) loading capacity, BMP_2_-modified black phosphorus (BP@BMP_2_) nanosheets are used to bind on a polylactic acid (PLLA) electrospun fibrous scaffold by microsol-electrospinning technique, realizing successfully a bioinspired staged bone regeneration strategy (Figure 5C) (Cheng et al., 2020). On this basis, BMP-2 can recruit pre-osteoblasts and promote their differentiation. Phosphate generated from BP also chelates Ca^+^ ions to deposit on electrospun fibrous scaffolds in a 3D manner. At last, P-BP@BMP2 nanofibrous scaffolds exhibit excellent bone regeneration ability.

A recent study has also found that the binding of extracellular vesicles (EVs) to black phosphorus can regulate intercellular communication to promote bone regeneration. In 2019, wang et al. engineered matrix bioinspired matrix vesicles (MVs), termed Apt-bioinspired MVs, through the intercalation of black phosphorus and functionalization of cell-specific aptamers (Apt) (Wang Y. et al., 2019). MVs, as a kind of EVs, are involved in the regulation of mineralization in the body (Hasegawa, 2018). Apt-bioinspired MVs can be directed to osteoblasts in bone tissue under the guidance of the aptamer and take advantage of the photothermal effect of black phosphorus to achieve the up-regulation of heat shock proteins and alkaline phosphatase. Simultaneously, the degradation product phosphate, as a component of bone, from BP can also promote the biomineralization process as a component of bone.

## 6 Other bio-materials

With the continuous progress of physical and chemical processing, other innovative smart multi-functional materials provided with both structural and therapeutical properties have been also discovered and prepared (Ambrosio et al., 2021). These biomaterials, such as bio-ceramics, molybdenum disulfide (MoS_2_), selenium (Se), natural polymers, etc., have also received more and more attention in the process of bone reconstruction after bone tumor surgery due to excellent osteogenesis-promoting ability, biodegradability, drug-loading ability, and anti-tumor effect of phototherapy, etc. (Table 2) (Turnbull et al., 2018; Koons et al., 2020).

**TABLE 2:** The main features of other bio-materials.

Type of bio-materials	Strengths	Flaws	Instance
Bio-ceramics	Corrosion resistance; good biocompatibility; bioactive ions, pressure resistance	Excessive brittleness	Calcium silicate CaCO_3_-PCL scaffold
MoS_2_	two-dimensional surface area; high near-infrared strong absorbance	Potential toxicity	MoS2-HA-DTPA-Gd/Gef
Selenium	Thermal and chemical stability	Acute or chronic poisoning; lack of targeting	Se-doped HA scaffolds Se-CaP
Natural polymers	Mimicking extracellular matrix; no toxicity	Potential immunogenicity; lower mechanical properties	Chitosan Curcumin Alginate

Bio-ceramics can release lots of bioactive ions, such as calcium ions, copper ions, silicon ions, magnesium ions, etc., to play the role of promoting osteogenesis and angiogenesis (Hoppe et al., 2011; Jones, 2013). In addition, some new breakthroughs have been made in bio-ceramics as a repair material for bone defects after osteosarcoma surgery recently (Chen et al., 2019; Elfeky et al., 2020; Yang et al., 2020). Firstly, bio-ceramics tend to mimic the extracellular matrix during bone tissue regeneration by providing mechanical support and an appropriate environment for mesenchymal stem cell attachment, proliferation, and differentiation (Shin et al., 2003; Kim et al., 2017). Besides, the bio-ceramic scaffold, functionalized with appropriate materials, also has the effect of photothermal anti-tumor. Meanwhile, bio-ceramic-based composites have important roles in anticancer drug delivery systems, including the treatment of osteosarcoma (Liu Y. et al., 2021; Oliveira et al., 2021). For, example, a 3D-printed calcium silicate material, after high photothermal functionalization, has the functions of anti-osteosarcoma, promoting bone regeneration and drug loading (Truong et al., 2021). On this basis, He et al. designed a 3D printed polymeric polycaprolactone fibers coated with porous calcium carbonate structures (PCL/CaCO3) scaffold and surface-modified it with 2D inorganic Egyptian blue nanosheets (CaPCu) (He et al., 2021). Here, Egyptian blue (EB, CaCuSi_4_O_10_), one of the oldest synthetic pigments containing silicon, copper, and calcium, has been revealed in previous studies to promote osteogenesis. To conclude, compared with other bone repair materials, the scaffold has greater advantages in terms of stronger photothermal ability under NIR-II laser irradiation, synergistic osteogenesis and antitumor ability, and orthotopic transplantation. Finally, bio-ceramics, as a biomaterial with both anti-tumor and promotion of bone repair, have also made some progress in the study of its mechanism. For instance, several reports have also found that nanoscale hydroxyapatite can inhibit the proliferation and migration of osteosarcoma by down-regulating the FAK/PI3K/Akt signaling pathways *in vitro* and *in vivo* (Wang R. et al., 2020). However, despite the advantages of bio-ceramics in high compressive modulus and provision of bioactive ions, the excessive brittleness limits further clinical applications. Therefore, the synthesis of potentially tough bio-ceramic polymer hybrids may help to overcome this challenge.

Beyond graphene and BP nanosheets, a new 2D material, MoS_2_, is attracting researchers’ attention due to its unique visible photoluminescence with high absorption (Shi et al., 2020). MoS_2_ disulfide has been reported to exhibit an indirect-to-direct semiconducting transition in the exfoliation from bulk to monolayer, which has led to the widespread use of MoS_2_ in electronic devices and catalysts (Voiry et al., 2016). More importantly, the application of MoS_2_ in the biomedical field has been continuously explored due to its large two-dimensional surface area and high near-infrared strong absorbance (Chou et al., 2013). For example, by combining MoS_2_ with hyaluronic acid, the instability of MoS_2_ and the low efficiency of the PTT in tissues are overcome (Shin et al., 2019). Meanwhile, hyaluronic acid promotes the accumulation of MoS_2_ in tumor cells through its mediated endocytosis, which enhances the efficiency of PTT and PA imaging of tumors (Lemaster and Jokerst, 2017; Fu et al., 2019). On this basis, Liu J. et al. (2019) designed a nanoplatform based on MoS_2_ and functionalized with hyaluronic acid for tumor MRI and synergistic chemo-photothermal therapy. This nanoplatform enables co-targeted delivery of gadolinium (Gd) based contrast agents and gefitinib (Gef). Both *in vitro* and *in vivo* experiments have demonstrated that under near-infrared radiation, MoS2-HA-DTPA-Gd/Gef can induce tumor cell apoptosis through the phosphatidylinositol 3 kinase (PI3K)/protein kinase B (Akt) signaling pathway, which provides new ideas for tumor diagnosis and treatment. Unfortunately, reports on the application of MoS_2_ in bone reconstruction after osteosarcoma surgery are rare. Besides, MoS_2_, as a transition metal dihalide, has a non-negligible low toxicity (Chen et al., 2018). In general, based on its unique properties, the application of MoS_2_ in the treatment and diagnosis of bone tumors deserves further exploration.

Selenium (Se), as one of the essential trace elements in the human body and a cofactor for dozens of enzymes in the body, is an indispensable part of the body’s oxidation, stress, immunity, and other reaction (Prabhu and Lei, 2016). Several previous reports have revealed the toxic effects of Se in various tumors, such as colon cancer, prostate cancer, breast cancer, etc. (Pang and Chin, 2019). Recently, it has also been reported that Se can improve the multidrug resistance (MDR) of osteosarcoma cells by inducing apoptosis in osteosarcoma treatment. For instance, Wang et al. found that selenium-doped nano-hydroxyapatite (Se-HANs) could exert an antitumor effect through the synergistic effect of caspase-dependent apoptosis and ROS-induced apoptosis (Li X. et al., 2020). On this basis, selenium-doped calcium phosphate (Se-CaP) organisms were engineered to carry the chemotherapeutic drug doxorubicin to target doxorubicin-resistant osteosarcoma cell line MG63/DXR in a xenografted BALB/c nude mice (Hu et al., 2021). In addition to caspase-dependent apoptosis and ROS-induced apoptosis, Se-CaP can downregulate the expression of MDR-associated ATP-binding cassette (ABC) transporters (ABCB1 and ABCC1) to reverse MDR (Hu et al., 2021). Besides, an article found that compared with hydroxyapatite, Se-doped hydroxyapatite scaffolds can synergistically promote the differentiation of human adipose-derived mesenchymal stem cells (hAD-MSCs) into bone tissue, thereby enhancing ALP activity and osteogenesis (Zakhireh et al., 2021). Furthermore, Li et al. found that the porous Se@SiO2 nanocomposite avoided the apoptosis of H_2_O_2_ on BMSCs through the BMP/SMAD pathway and promoted the osteogenic differentiation of BMSCs (Li C. et al., 2019). Overall, Se-doped bio-scaffolds may offer new clinical benefits for bone tissue engineering (Zeng et al., 2013). However, although Se deficiency may be associated with some biological disturbances, Se can also cause acute or chronic poisoning, often manifesting as brittle and falling nails, gastrointestinal disturbances, rashes, fatigue, irritability, and neurological abnormalities (Cao et al., 2012; Cardoso et al., 2021). Therefore, the design of Se-doped scaffolds for sustained release of Se to obtain a safe concentration in the body may be the focus of future research.

Natural polymers, such as chitosan, curcumin, alginate, etc., have received a lot of attention in bone tissue engineering due to their advantages of mimicking ECM, providing cell adhesion sites, and low cost. Recently, unlike previous studies, an article has revealed that extracellular matrix elasticity, rather than matrix adherence, modulates tumor cell growth through integrin-mediated focal adhesion (FA) signaling (Chaudhuri et al., 2014; Jiang et al., 2019). In contrast, normal cells, such as osteoblasts, are primarily affected by ECM adhesion ligands through integrin-mediated regulation of the adherens junction (AJ) signaling pathway (Steinbacher and Ebnet, 2018). This provides new insights into bone tissue engineering. Based on this, Tan et al. designed an injectable curcumin microsphere/IR820 hybrid bifunctional hydrogel, which can not only play the role of photothermal anti-tumor but also promote bone remodeling through the sustained release of curcumin (Tan et al., 2021). Meanwhile, the heat generated by PTT *in vivo* accelerates the release of curcumin and induces apoptosis of osteosarcoma cells. In addition, studies have confirmed that 3D-printed hydroxyapatite scaffolds added with chitosan can further promote the development of new bone tissue *in vitro* and vivo, which is beneficial to osseointegration (Zafeiris et al., 2021). The degradability of the scaffolds will benefit patients in terms of improved quality of life while avoiding some complications. However, although natural polymers are more and more widely used as multifunctional materials in bone defects after osteosarcoma surgery, their existing problems, such as potential immunogenicity and lower mechanical properties, should also be paid attention to several studies have made progress on the lack of rigidity by 3D-printing hybrid scaffolds made by combining natural polymers with bio-ceramics. But this is not enough, more modified natural polymers are needed for better clinical application.

Recently, ferroptosis as a form of regulated cell death has also attracted great interest in tumor research (Dixon et al., 2012; Zhang et al., 2022). The earliest studies found that ferroptosis is mainly caused by an iron-dependent accumulation of lipid peroxidation, inactivation/depletion of anti-lipid peroxidation molecules, and increased mitochondrial membrane density. On this basis, several studies have made some progress in the non-surgical treatment of tumors by inducing ferroptosis of tumor cells through biomaterials (Yang et al., 2021d; Han et al., 2022). For example, Fu et al. (2021) designed a mesoporous silica nanoplatform integrating doxorubicin and ferrate by assembling a solid-liquid phase change material of *n*-heneicosane, thus realizing the co-release of doxorubicin and ferrate under ultrasound (US). Surprisingly, exogenous iron derived from the metabolism of this nanodrug can induce ferroptosis in tumor cells and exert a synergistic anti-tumor effect. Furthermore, an article found that singlet oxygen generated by photodynamic therapy of nanomaterials can promote the ferroptosis of tumor cells (Li J. et al., 2021). In conclusion, combining photothermal therapy, photodynamic therapy, and ferroptosis with nanomaterials provides a new perspective for dealing with the issue of tumor tissue recurrence after osteosarcoma surgery and the chemoresistance of osteosarcoma. However, the current problem is that studies on ferroptosis in osteosarcoma are rare. Therefore, new explorations based on nano-biomaterials are highly required to deal with the challenges of killing residual tumor cells and bone remodeling after osteosarcoma surgery.

## 7 Prospects and clinical translation

With excellent anti-tumor and bone-promoting effects, the above biomaterials have become a hot spot in the current spotlight, and have also been preliminaries explored in clinical translational research. Then, we briefly describe the prospects and clinical translation of magnesium, silver and black phosphorus.

### 7.1 Magnesium

Mg has been regarded as a promising bioactive material for bone regeneration due to its sufficient mechanical properties, biodegradability and osteogenic activity. But in fact, the application of pure Mg implants still faces some challenges, such as rapid degradation, excessive hydrogen formation, and the difficulty of fabricating magnesium-based multi-pore scaffolds.

Encouragingly, the use of orthopedic devices or implants, for instance screws based on magnesium or its alloys in fracture repair has been reported in China and Germany in recent years, with promising osteogenic results compared to titanium (Windhagen et al., 2013; Zhao et al., 2016; Zhao et al., 2017). Besides, as mentioned earlier, magnesium particles are integrated into biodegradable polymer substrates such as PLGA to create composite scaffolds (PLGA/Mg) to circumvent these defects (Long et al., 2021). On the other hand, the surface modification of Mg alloy, enhancing the corrosion resistance and mechanical strength of Mg metal and avoiding a large amount of local hydrogen accumulation, can also pave the way for the clinical translation of Mg implants (Gao et al., 2021).

Less noticed but as important, magnetic hyperthermia (MHT) was found to be able to ablate the tumor using an alternating magnetic field (AMF) to heat a magnetothermal agent (magnetic nanoparticles including magnesium particles) applied to the tumor (Yang N. et al., 2021). The potential interaction between human biological magnetic field and this MHT may bring different prospects for clinical application of Mg materials.

Mg-induced osteogenesis is mediated by local neuronal production of calcitonin gene-related peptide 1 (CGRP1) and has been demonstrated in fractured mice suggesting that magnesium may be involved in the neural regulation of bone defects and thus in the regulation of bone homeostasis (Zhang Y. et al., 2016). This means that other roles of magnesium in the process of bone reconstruction after bone tumor surgery, such as pain regulation and angiogenesis, are also worth further investigation.

### 7.2 Silver

As described above, silver has been used in our clinical practice for hundreds of years, from antibacterial and regenerative to today’s anti-tumor. At present, the academic research mainly focuses on nanoscale silver particles, such as AgNP and AgAP, due to their excellent tumor targeting, killing effects and potential osteogenesis.

Consequently, current clinical studies aim to improve cancer treatment by modifying Ag NPs to track and specifically bind tumor cells *in vivo*, thereby improving cancer treatment with minimal risk to normal cells. Beyond that, the exploration of different nanoparticle shapes for optimal drug delivery is the focus of current clinical research (Malik and Mukherjee, 2018). But regrettably, this part of clinical research is lacking in osteosarcoma. Therefore, the potential widespread application of silver nanoparticles in preclinical and clinical stages of osteosarcoma should not stop exploration, although the current research and development is still in its infancy and face many difficulties.

### 7.3 Black phosphorus

Black phosphorus not only can be completely degraded into non-toxic phosphate, but also can fully kill tumors through phototherapy, making it a favorite in the field of biomaterials, especially in the field of bone tumors.

The broad prospect of clinical translational application of BP has attracted the attention of researchers mainly because of its unique pre-osteogenic ability in the field of bone repair. For example, chitosan thermal response hydrogel therapy can be used to treat bone defects caused by arthritis rheumatoid arthritis (RA) by adding BP nanosheets to platelet-rich plasma (PRP) (Pan et al., 2020). Yet, in the case of osteosarcoma, the clinical translation of black phosphorus-based biomaterials is still in the infant segment, despite ongoing exploration of the immune and metabolic microenvironment during bone defect reconstruction. Fortunately, black phosphorus, with its high electrical conductivity, seems to be a breakthrough point for clinical translational research by participating in nerve fiber repair to promote bone regeneration (Grassel, 2014; Qian et al., 2019). In conclusion, the clinical application prospect of BP can be predicted, based on its three major properties of promoting *in situ* mineralization through degradation product phosphate, inducing nerve regeneration and regulating bone repair, and photothermal treatment killing tumor although the mainstream research of black phosphorus in osteosarcoma is still concentrated in the experimental stage.

## 8 Summary and discussion

This article introduces biomaterials of recent years, such as black phosphorus, magnesium, zinc, copper, silver, etc., with their good biocompatibility, biodegradation antibacterial, and anti-tumor effects, they have received high attention and consideration from researchers. Many studies have been successful in cell or animal experiments. However, in looking for an ideal material that can not only fill in but also kill the residual tumor cells, reducing the probability of recurrence and metastasis, and hence promoting bone repair, there is still a long way to go in the clinical treatment of osteosarcoma.

Overall, magnesium-zinc alloys and copper are relatively under-studied, while silver and black phosphorus are relatively studied and are on the rise due to their various functions and safe use. Additionally, MoS_2_ and bio-ceramics, etc. have also attracted a lot of interest in bone tissue engineering. Above all, for the study of silver, the advantage is that it has anti-tumor and antibacterial effects, but there are few studies on long-term toxic and side effects, and it is worth continuing to explore. At the same time, currently, the targeted therapy of AgNPs and AgAPs is mainly targeting the acidic environment of tumors through the effect of EPR, so more accurate targeted therapy for tumors is urgently needed. For black phosphorus, the biologically active phosphorus-based drug therapy has just started, and the specific molecular mechanism may be a direction worth exploring. However, although a controlled degradation mode can be obtained by irradiation with near infrared light, how to improve the targeting of black phosphorus nanomaterials is also a clinical problem. But targeted therapy based on specific molecules on the surface of the osteosarcoma may help improve targeting although osteosarcoma is a highly heterogeneous tumor. Secondly, for zinc, magnesium, and copper, the rapid development of nanotechnology has made them another breakthrough after being used as the substrate for 3D printing scaffolds. Among them, potential toxicity and rapid degradation of zinc limit its application in bone reconstruction after osteosarcoma surgery. However, combining zinc coating with 3D-printed scaffolds, such as PLGA, BG may produce unexpected applications. Due to excellent osteogenic activity and surface modification, Mg has made great strides in clinical conversion applications, despite its rapid degradation rate and excessive local hydrogen production. In the meantime, the research on the MHT and neural regulation of magnesium has opened up a new direction for the study of magnesium. Then, MoS_2_, a discovery of two-dimensional materials after black phosphorus nanosheets, possesses excellent anti-tumor effects and photoacoustic imaging capabilities. However, research in osteosarcoma is rare, and its application in osteosarcoma deserves further exploration. Finally, the research of modified polymer compounds and selenium in bone tissue engineering has also attained a new turning point.

In general, we consider the latest application of biomaterials in bone reconstruction after osteosarcoma surgery as a remedy for large bone defects after osteosarcoma surgery as well as recurrence and metastasis caused by residual tumor tissue. At the same time, advances in nanomaterials have enabled the better use of phototherapy, tumor imaging, and targeted drug delivery. It is expected that this will inspire future research to bring further developments in the treatment of patients with osteosarcoma.

## References

[B1] Alzeibak R. Mishchenko T. A. Shilyagina N. Y. Balalaeva I. V. Vedunova M. V. Krysko D. V. (2021). Targeting immunogenic cancer cell death by photodynamic therapy: Past, present and future. J. Immunother. Cancer 9 (1), e001926. 10.1136/jitc-2020-001926 33431631 PMC7802670

[B2] Ambrosio L. Raucci M. G. Vadala G. Ambrosio L. Papalia R. Denaro V. (2021). Innovative biomaterials for the treatment of bone cancer. Int. J. Mol. Sci. 22 (15), 8214. 10.3390/ijms22158214 34360979 PMC8347125

[B3] Ameh T. Sayes C. M. (2019). The potential exposure and hazards of copper nanoparticles: A review. Environ. Toxicol. Pharmacol. 71, 103220. 10.1016/j.etap.2019.103220 31306862

[B4] Biju V. (2014). Chemical modifications and bioconjugate reactions of nanomaterials for sensing, imaging, drug delivery and therapy. Chem. Soc. Rev. 43 (3), 744–764. 10.1039/c3cs60273g 24220322

[B5] Bonnet S. Archer S. L. Allalunis-Turner J. Haromy A. Beaulieu C. Thompson R. (2007). A mitochondria-K+ channel axis is suppressed in cancer and its normalization promotes apoptosis and inhibits cancer growth. Cancer Cell 11 (1), 37–51. 10.1016/j.ccr.2006.10.020 17222789

[B6] Brewer G. J. (2010). Risks of copper and iron toxicity during aging in humans. Chem. Res. Toxicol. 23 (2), 319–326. 10.1021/tx900338d 19968254

[B7] Cao J. J. Gregoire B. R. Zeng H. (2012). Selenium deficiency decreases antioxidative capacity and is detrimental to bone microarchitecture in mice. J. Nutr. 142 (8), 1526–1531. 10.3945/jn.111.157040 22739365

[B8] Cardoso B. R. Cominetti C. Seale L. A. (2021). Editorial: Selenium, human health and chronic disease. Front. Nutr. 8, 827759. 10.3389/fnut.2021.827759 35118114 PMC8803728

[B9] Chaudhuri O. Koshy S. T. Branco da Cunha C. Shin J. W. Verbeke C. S. Allison K. H. (2014). Extracellular matrix stiffness and composition jointly regulate the induction of malignant phenotypes in mammary epithelium. Nat. Mater 13 (10), 970–978. 10.1038/nmat4009 24930031

[B10] Chen C. Y. Yin H. Chen X. Chen T. H. Liu H. M. Rao S. S. (2020). Angstrom-scale silver particle-embedded carbomer gel promotes wound healing by inhibiting bacterial colonization and inflammation. Sci. Adv. 6 (43), eaba0942. 10.1126/sciadv.aba0942 33097529 PMC7608828

[B11] Chen E. Xue D. Zhang W. Lin F. Pan Z. (2015). Extracellular heat shock protein 70 promotes osteogenesis of human mesenchymal stem cells through activation of the ERK signaling pathway. FEBS Lett. 589 (24), 4088–4096. 10.1016/j.febslet.2015.11.021 26608032

[B12] Chen L. Deng C. Li J. Yao Q. Chang J. Wang L. (2019). 3D printing of a lithium-calcium-silicate crystal bioscaffold with dual bioactivities for osteochondral interface reconstruction. Biomaterials 196, 138–150. 10.1016/j.biomaterials.2018.04.005 29643002

[B13] Chen W. Qi W. Lu W. Chaudhury N. R. Yuan J. Qin L. (2018). Direct assessment of the toxicity of molybdenum disulfide atomically thin film and microparticles via cytotoxicity and patch testing. Small 14 (12), e1702600. 10.1002/smll.201702600 29356309

[B14] Chen Z. Meng H. Xing G. Chen C. Zhao Y. Jia G. (2006). Acute toxicological effects of copper nanoparticles *in vivo* . Toxicol. Lett. 163 (2), 109–120. 10.1016/j.toxlet.2005.10.003 16289865

[B15] Cheng L. Chen Z. Cai Z. Zhao J. Lu M. Liang J. (2020). Bioinspired functional black phosphorus electrospun fibers achieving recruitment and biomineralization for staged bone regeneration. Small 16 (50), e2005433. 10.1002/smll.202005433 33230977

[B16] Choi J. R. Yong K. W. Choi J. Y. Nilghaz A. Lin Y. Xu J. (2018). Black phosphorus and its biomedical applications. Theranostics 8 (4), 1005–1026. 10.7150/thno.22573 29463996 PMC5817107

[B17] Chou S. S. Kaehr B. Kim J. Foley B. M. De M. Hopkins P. E. (2013). Chemically exfoliated MoS2 as near-infrared photothermal agents. Angew. Chem. Int. Ed. Engl. 52 (15), 4160–4164. 10.1002/anie.201209229 23471666 PMC4193793

[B18] Chugh H. Sood D. Chandra I. Tomar V. Dhawan G. Chandra R. (2018). Role of gold and silver nanoparticles in cancer nano-medicine. Artif. Cells Nanomed Biotechnol. 46 (1), 1210–1220. 10.1080/21691401.2018.1449118 29533101

[B19] Dang W. Ma B. Li B. Huan Z. Ma N. Zhu H. (2020). 3D printing of metal-organic framework nanosheets-structured scaffolds with tumor therapy and bone construction. Biofabrication 12 (2), 025005. 10.1088/1758-5090/ab5ae3 31756727

[B20] Diez-Tercero L. Delgado L. M. Bosch-Rue E. Perez R. A. (2021). Evaluation of the immunomodulatory effects of cobalt, copper and magnesium ions in a pro inflammatory environment. Sci. Rep. 11 (1), 11707. 10.1038/s41598-021-91070-0 34083604 PMC8175577

[B21] Dixon S. J. Lemberg K. M. Lamprecht M. R. Skouta R. Zaitsev E. M. Gleason C. E. (2012). Ferroptosis: An iron-dependent form of nonapoptotic cell death. Cell 149 (5), 1060–1072. 10.1016/j.cell.2012.03.042 22632970 PMC3367386

[B22] Dziedzic A. Kubina R. Buldak R. J. Skonieczna M. Cholewa K. (2016). Silver nanoparticles exhibit the dose-dependent anti-proliferative effect against human squamous carcinoma cells attenuated in the presence of berberine. Molecules 21 (3), 365. 10.3390/molecules21030365 26999092 PMC6274313

[B23] Elfeky S. A. Elsayed A. Moawad M. Ahmed W. A. (2020). Hydroxyapatite nanocomposite as a potential agent in osteosarcoma PDT. Photodiagnosis Photodyn. Ther. 32, 102056. 10.1016/j.pdpdt.2020.102056 33068821

[B24] Fernandes M. H. Alves M. M. Cebotarenco M. Ribeiro I. A. C. Grenho L. Gomes P. S. (2020). Citrate zinc hydroxyapatite nanorods with enhanced cytocompatibility and osteogenesis for bone regeneration. Mater Sci. Eng. C Mater Biol. Appl. 115, 111147. 10.1016/j.msec.2020.111147 32600733

[B25] Ferrari S. Bacci G. Picci P. Mercuri M. Briccoli A. Pinto D. (1997). Long-term follow-up and post-relapse survival in patients with non-metastatic osteosarcoma of the extremity treated with neoadjuvant chemotherapy. Ann. Oncol. 8 (8), 765–771. 10.1023/a:1008221713505 9332684

[B26] Fu J. Li T. Yang Y. Jiang L. Wang W. Fu L. (2021). Activatable nanomedicine for overcoming hypoxia-induced resistance to chemotherapy and inhibiting tumor growth by inducing collaborative apoptosis and ferroptosis in solid tumors. Biomaterials 268, 120537. 10.1016/j.biomaterials.2020.120537 33260096

[B27] Fu Q. Zhu R. Song J. Yang H. Chen X. (2019). Photoacoustic imaging: Contrast agents and their biomedical applications. Adv. Mater 31 (6), e1805875. 10.1002/adma.201805875 30556205

[B28] Gao J. Su Y. Qin Y. X. (2021). Calcium phosphate coatings enhance biocompatibility and degradation resistance of magnesium alloy: Correlating *in vitro* and *in vivo* studies. Bioact. Mater 6 (5), 1223–1229. 10.1016/j.bioactmat.2020.10.024 33210020 PMC7653207

[B29] Gao L. C. Q. Gong T. Liu J. Li C. (2019). “Ecent advancement of imidazolate framework (ZIF-8) based nanoformulations for synergistic tumor therapy,” in Nanoscale. 10.1039/c9nr06558j 31674617

[B30] Ge X. Wong R. Anisa A. Ma S. (2022). Recent development of metal-organic framework nanocomposites for biomedical applications. Biomaterials 281, 121322. 10.1016/j.biomaterials.2021.121322 34959029

[B31] Geng S. Pan T. Zhou W. Cui H. Wu L. Li Z. (2020). Bioactive phospho-therapy with black phosphorus for *in vivo* tumor suppression. Theranostics 10 (11), 4720–4736. 10.7150/thno.43092 32308745 PMC7163432

[B32] Gill J. Gorlick R. (2021). Advancing therapy for osteosarcoma. Nat. Rev. Clin. Oncol. 18 (10), 609–624. 10.1038/s41571-021-00519-8 34131316

[B33] Grassel S. G. (2014). The role of peripheral nerve fibers and their neurotransmitters in cartilage and bone physiology and pathophysiology. Arthritis Res. Ther. 16 (6), 485. 10.1186/s13075-014-0485-1 25789373 PMC4395972

[B34] Gui R. Jin H. Wang Z. Li J. (2018). Black phosphorus quantum dots: Synthesis, properties, functionalized modification and applications. Chem. Soc. Rev. 47 (17), 6795–6823. 10.1039/c8cs00387d 30014059

[B35] Han W. Duan X. Ni K. Li Y. Chan C. Lin W. (2022). Co-delivery of dihydroartemisinin and pyropheophorbide-iron elicits ferroptosis to potentiate cancer immunotherapy. Biomaterials 280, 121315. 10.1016/j.biomaterials.2021.121315 34920370 PMC8724418

[B36] Han X. He J. Wang Z. Bai Z. Qu P. Song Z. (2021). Fabrication of silver nanoparticles/gelatin hydrogel system for bone regeneration and fracture treatment. Drug Deliv. 28 (1), 319–324. 10.1080/10717544.2020.1869865 33517806 PMC8725951

[B37] Harris E. D. (1992). Copper as a cofactor and regulator of copper, zinc superoxide dismutase. J. Nutr. 122 (3), 636–640. 10.1093/jn/122.suppl_3.636 1542024

[B38] Hasegawa T. (2018). Ultrastructure and biological function of matrix vesicles in bone mineralization. Histochem Cell Biol. 149 (4), 289–304. 10.1007/s00418-018-1646-0 29411103

[B39] He C. Dong C. Yu L. Chen Y. Hao Y. (2021). Ultrathin 2D inorganic ancient pigment decorated 3D-printing scaffold enables photonic hyperthermia of osteosarcoma in NIR-II biowindow and concurrently augments bone regeneration. Adv. Sci. (Weinh) 8 (19), e2101739. 10.1002/advs.202101739 34338444 PMC8498872

[B40] He G. Ma Y. Zhu Y. Yong L. Liu X. Wang P. (2018). Cross talk between autophagy and apoptosis contributes to ZnO nanoparticle-induced human osteosarcoma cell death. Adv. Healthc. Mater 7 (17), e1800332. 10.1002/adhm.201800332 29900694 PMC6310009

[B41] He G. Pan X. Liu X. Zhu Y. Ma Y. Du C. (2020). HIF-1α-Mediated mitophagy determines ZnO nanoparticle-induced human osteosarcoma cell death both *in vitro* and *in vivo* . ACS Appl. Mater Interfaces 12 (43), 48296–48309. 10.1021/acsami.0c12139 33054172

[B42] He P. Xu S. Guo Z. Yuan P. Liu Y. Chen Y. (2022). Pharmacodynamics and pharmacokinetics of PLGA-based doxorubicin-loaded implants for tumor therapy. Drug Deliv. 29 (1), 478–488. 10.1080/10717544.2022.2032878 35147071 PMC8843208

[B43] Heng M. Gupta A. Chung P. W. Healey J. H. Vaynrub M. Rose P. S. (2020). The role of chemotherapy and radiotherapy in localized extraskeletal osteosarcoma. Eur. J. Cancer 125, 130–141. 10.1016/j.ejca.2019.07.029 31806415 PMC7261507

[B44] Hoppe A. Güldal N. S. Boccaccini A. R. (2011). A review of the biological response to ionic dissolution products from bioactive glasses and glass-ceramics. Biomaterials 32 (11), 2757–2774. 10.1016/j.biomaterials.2011.01.004 21292319

[B45] Hou X. Tao Y. Pang Y. Li X. Jiang G. Liu Y. (2018). Nanoparticle-based photothermal and photodynamic immunotherapy for tumor treatment. Int. J. Cancer 143 (12), 3050–3060. 10.1002/ijc.31717 29981170

[B46] Hu J. Jiang Y. Tan S. Zhu K. Cai T. Zhan T. (2021). Selenium-doped calcium phosphate biomineral reverses multidrug resistance to enhance bone tumor chemotherapy. Nanomedicine 32, 102322. 10.1016/j.nano.2020.102322 33186694

[B47] Hu X. K. Rao S. S. Tan Y. J. Yin H. Luo M. J. Wang Z. X. (2020). Fructose-coated Angstrom silver inhibits osteosarcoma growth and metastasis via promoting ROS-dependent apoptosis through the alteration of glucose metabolism by inhibiting PDK. Theranostics 10 (17), 7710–7729. 10.7150/thno.45858 32685015 PMC7359101

[B48] Huang T. Yan G. Guan M. (2020). Zinc homeostasis in bone: Zinc transporters and bone diseases. Int. J. Mol. Sci. 21 (4), 1236. 10.3390/ijms21041236 32059605 PMC7072862

[B49] Jiang T. Xu G. Chen X. Huang X. Zhao J. Zheng L. (2019). Impact of hydrogel elasticity and adherence on osteosarcoma cells and osteoblasts. Adv. Healthc. Mater 8 (9), e1801587. 10.1002/adhm.201801587 30838809

[B50] Jiang Z. Li T. Cheng H. Zhang F. Yang X. Wang S. (2021). Nanomedicine potentiates mild photothermal therapy for tumor ablation. Asian J. Pharm. Sci. 16 (6), 738–761. 10.1016/j.ajps.2021.10.001 35027951 PMC8739255

[B51] Jo D. H. Kim J. H. Lee T. G. Kim J. H. (2015). Size, surface charge, and shape determine therapeutic effects of nanoparticles on brain and retinal diseases. Nanomedicine 11 (7), 1603–1611. 10.1016/j.nano.2015.04.015 25989200

[B52] Jones J. R. (2013). Review of bioactive glass: From hench to hybrids. Acta Biomater. 9 (1), 4457–4486. 10.1016/j.actbio.2012.08.023 22922331

[B53] Kargozar S. Mozafari M. Ghodrat S. Fiume E. Baino F. (2021). Copper-containing bioactive glasses and glass-ceramics: From tissue regeneration to cancer therapeutic strategies. Mater Sci. Eng. C Mater Biol. Appl. 121, 111741. 10.1016/j.msec.2020.111741 33579436

[B54] Kim H. D. Amirthalingam S. Kim S. L. Lee S. S. Rangasamy J. Hwang N. S. (2017). Biomimetic materials and fabrication approaches for bone tissue engineering. Adv. Healthc. Mater 6 (23), 1700612. 10.1002/adhm.201700612 29171714

[B55] Koons G. L. Diba M. Mikos A. G. (2020). Materials design for bone-tissue engineering. Nat. Rev. Mater. 5 (8), 584–603. 10.1038/s41578-020-0204-2

[B56] Kou L. Chen C. Smith S. C. (2015). Phosphorene: Fabrication, properties, and applications. J. Phys. Chem. Lett. 6 (14), 2794–2805. 10.1021/acs.jpclett.5b01094 26266865

[B57] Kovacs D. Igaz N. Keskeny C. Belteky P. Toth T. Gaspar R. (2016). Silver nanoparticles defeat p53-positive and p53-negative osteosarcoma cells by triggering mitochondrial stress and apoptosis. Sci. Rep. 6, 27902. 10.1038/srep27902 27291325 PMC4904210

[B58] Krol A. Pomastowski P. Rafinska K. Railean-Plugaru V. Buszewski B. (2017). Zinc oxide nanoparticles: Synthesis, antiseptic activity and toxicity mechanism. Adv. Colloid Interface Sci. 249, 37–52. 10.1016/j.cis.2017.07.033 28923702

[B59] Lemaster J. E. Jokerst J. V. (2017). What is new in nanoparticle-based photoacoustic imaging? Wiley Interdiscip. Rev. Nanomed Nanobiotechnol 9 (1). 10.1002/wnan.1404 PMC5045757 27038222

[B60] Li C. Wang Q. Gu X. Kang Y. Zhang Y. Hu Y. (2019). Porous Se@SiO2 nanocomposite promotes migration and osteogenic differentiation of rat bone marrow mesenchymal stem cell to accelerate bone fracture healing in a rat model. Int. J. Nanomedicine 14, 3845–3860. 10.2147/IJN.S202741 31213805 PMC6539174

[B61] Li J. Li J. Pu Y. Li S. Gao W. He B. (2021). PDT-enhanced ferroptosis by a polymer nanoparticle with pH-activated singlet oxygen generation and superb biocompatibility for cancer therapy. Biomacromolecules 22 (3), 1167–1176. 10.1021/acs.biomac.0c01679 33566577

[B62] Li S. Sun W. Wang H. Zuo D. Hua Y. Cai Z. (2015). Research progress on the multidrug resistance mechanisms of osteosarcoma chemotherapy and reversal. Tumour Biol. 36 (3), 1329–1338. 10.1007/s13277-015-3181-0 25666750

[B63] Li W W. Yang J. Luo L. Jiang M. Qin B. Yin H. (2019). Targeting photodynamic and photothermal therapy to the endoplasmic reticulum enhances immunogenic cancer cell death. Nat. Commun. 10 (1), 3349. 10.1038/s41467-019-11269-8 31350406 PMC6659660

[B64] Li X. Wang Y. Chen Y. Zhou P. Wei K. Wang H. (2020). Hierarchically constructed selenium-doped bone-mimetic nanoparticles promote ROS-mediated autophagy and apoptosis for bone tumor inhibition. Biomaterials 257, 120253. 10.1016/j.biomaterials.2020.120253 32738660

[B65] Li Y. Xiong J. Guo W. Jin Y. Miao W. Wang C. (2021). Decomposable black phosphorus nano-assembly for controlled delivery of cisplatin and inhibition of breast cancer metastasis. J. Control Release 335, 59–74. 10.1016/j.jconrel.2021.05.013 33992704

[B66] Li Z. Fu Q. Ye J. Ge X. Wang J. Song J. (2020). Ag(+) -coupled black phosphorus vesicles with emerging NIR-II photoacoustic imaging performance for cancer immune-dynamic therapy and fast wound healing. Angew. Chem. Int. Ed. Engl. 59 (49), 22202–22209. 10.1002/anie.202009609 32841465

[B67] Li Z. Zhang X. Ouyang J. Chu D. Han F. Shi L. (2021). Ca(2+)-supplying black phosphorus-based scaffolds fabricated with microfluidic technology for osteogenesis. Bioact. Mater 6 (11), 4053–4064. 10.1016/j.bioactmat.2021.04.014 33997492 PMC8089774

[B68] Liao J. Han R. Wu Y. Qian Z. (2021). Review of a new bone tumor therapy strategy based on bifunctional biomaterials. Bone Res. 9 (1), 18. 10.1038/s41413-021-00139-z 33727543 PMC7966774

[B69] Liu J. Dong J. Zhang T. Peng Q. (2018). Graphene-based nanomaterials and their potentials in advanced drug delivery and cancer therapy. J. Control Release 286, 64–73. 10.1016/j.jconrel.2018.07.034 30031155

[B70] Liu J. Zheng J. Nie H. Zhang D. Cao D. Xing Z. (2019). Molybdenum disulfide-based hyaluronic acid-guided multifunctional theranostic nanoplatform for magnetic resonance imaging and synergetic chemo-photothermal therapy. J. Colloid Interface Sci. 548, 131–144. 10.1016/j.jcis.2019.04.022 30991180

[B71] Liu M M. O'Connor R. S. Trefely S. Graham K. Snyder N. W. Beatty G. L. (2019). Metabolic rewiring of macrophages by CpG potentiates clearance of cancer cells and overcomes tumor-expressed CD47-mediated 'don't-eat-me' signal. Nat. Immunol. 20 (3), 265–275. 10.1038/s41590-018-0292-y 30664738 PMC6380920

[B72] Liu W W. Dong A. Wang B. Zhang H. (2021). Current advances in black phosphorus-based drug delivery systems for cancer therapy. Adv. Sci. (Weinh) 8 (5), 2003033. 10.1002/advs.202003033 33717847 PMC7927632

[B73] Liu Y. Raina D. B. Sebastian S. Nagesh H. Isaksson H. Engellau J. (2021). Sustained and controlled delivery of doxorubicin from an *in-situ* setting biphasic hydroxyapatite carrier for local treatment of a highly proliferative human osteosarcoma. Acta Biomater. 131, 555–571. 10.1016/j.actbio.2021.07.016 34271171

[B74] Long J. Zhang W. Chen Y. Teng B. Liu B. Li H. (2021). Multifunctional magnesium incorporated scaffolds by 3D-Printing for comprehensive postsurgical management of osteosarcoma. Biomaterials 275, 120950. 10.1016/j.biomaterials.2021.120950 34119886

[B75] Ma H. He C. Cheng Y. Li D. Gong Y. Liu J. (2014). PLK1shRNA and doxorubicin co-loaded thermosensitive PLGA-PEG-PLGA hydrogels for osteosarcoma treatment. Biomaterials 35 (30), 8723–8734. 10.1016/j.biomaterials.2014.06.045 25017095

[B76] Ma L. Feng X. Liang H. Wang K. Song Y. Tan L. (2020). A novel photothermally controlled multifunctional scaffold for clinical treatment of osteosarcoma and tissue regeneration. Mater. Today 36, 48–62. 10.1016/j.mattod.2019.12.005

[B77] Maeda H. (2001). The enhanced permeability and retention (EPR) effect in tumor vasculature: The key role of tumor-selective macromolecular drug targeting. Adv. Enzyme Regul. 41 (1), 189–207. 10.1016/s0065-2571(00)00013-3 11384745

[B78] Malik P. Mukherjee T. K. (2018). Recent advances in gold and silver nanoparticle based therapies for lung and breast cancers. Int. J. Pharm. 553 (1-2), 483–509. 10.1016/j.ijpharm.2018.10.048 30394284

[B79] Mao C. Xiang Y. Liu X. Cui Z. Yang X. Li Z. (2018). Repeatable photodynamic therapy with triggered signaling pathways of fibroblast cell proliferation and differentiation to promote bacteria-accompanied wound healing. ACS Nano 12 (2), 1747–1759. 10.1021/acsnano.7b08500 29376340

[B80] Marques C. Ferreira J. M. Andronescu E. Ficai D. Sonmez M. Ficai A. (2014). Multifunctional materials for bone cancer treatment. Int. J. Nanomedicine 9, 2713–2725. 10.2147/IJN.S55943 24920907 PMC4044993

[B81] Meazza C. Scanagatta P. (2016). Metastatic osteosarcoma: A challenging multidisciplinary treatment. Expert Rev. Anticancer Ther. 16 (5), 543–556. 10.1586/14737140.2016.1168697 26999418

[B82] Mei Z. Gao D. Hu D. Zhou H. Ma T. Huang L. (2020). Activatable NIR-II photoacoustic imaging and photochemical synergistic therapy of MRSA infections using miniature Au/Ag nanorods. Biomaterials 251, 120092. 10.1016/j.biomaterials.2020.120092 32388165

[B83] Mendel R. R. Smith A. G. Marquet A. Warren M. J. (2007). Metal and cofactor insertion. Nat. Prod. Rep. 24 (5), 963–971. 10.1039/b703112m 17898892

[B84] Miao Y. Shi X. Li Q. Hao L. Liu L. Liu X. (2019). Engineering natural matrices with black phosphorus nanosheets to generate multi-functional therapeutic nanocomposite hydrogels. Biomater. Sci. 7 (10), 4046–4059. 10.1039/c9bm01072f 31435628

[B85] Mirabello L. Troisi R. J. Savage S. A. (2009). Osteosarcoma incidence and survival rates from 1973 to 2004: Data from the surveillance, epidemiology, and end results program. Cancer 115 (7), 1531–1543. 10.1002/cncr.24121 19197972 PMC2813207

[B86] Muscolo D. L. Ayerza M. A. Aponte-Tinao L. A. Ranalletta M. (2005). Partial epiphyseal preservation and intercalary allograft reconstruction in high-grade metaphyseal osteosarcoma of the knee. J. Bone Jt. Surg. Am. 87 (2), 226–236. 10.2106/JBJS.E.00253 16140796

[B87] Norgaard R. Kassem M. Rattan S. I. (2006). Heat shock-induced enhancement of osteoblastic differentiation of hTERT-immortalized mesenchymal stem cells. Ann. N. Y. Acad. Sci. 1067, 443–447. 10.1196/annals.1354.063 16804024

[B88] Oliveira T. M. Berti F. C. B. Gasoto S. C. Schneider B. Jr. Stimamiglio M. A. Berti L. F. (2021). Calcium phosphate-based bioceramics in the treatment of osteosarcoma: Drug delivery composites and magnetic hyperthermia agents. Front. Med. Technol. 3, 700266. 10.3389/fmedt.2021.700266 35047940 PMC8757807

[B89] Palacios C. (2006). The role of nutrients in bone health, from A to Z. Crit. Rev. Food Sci. Nutr. 46 (8), 621–628. 10.1080/10408390500466174 17092827

[B90] Pan W. Dai C. Li Y. Yin Y. Gong L. Machuki J. O. (2020). PRP-chitosan thermoresponsive hydrogel combined with black phosphorus nanosheets as injectable biomaterial for biotherapy and phototherapy treatment of rheumatoid arthritis. Biomaterials 239, 119851. 10.1016/j.biomaterials.2020.119851 32078955

[B91] Pang K. L. Chin K. Y. (2019). Emerging anticancer potentials of selenium on osteosarcoma. Int. J. Mol. Sci. 20 (21), 5318. 10.3390/ijms20215318 31731474 PMC6862058

[B92] Poon W. Zhang Y. N. Ouyang B. Kingston B. R. Wu J. L. Y. Wilhelm S. (2019). Elimination pathways of nanoparticles. ACS Nano 13 (5), 5785–5798. 10.1021/acsnano.9b01383 30990673

[B93] Prabhu K. S. Lei X. G. (2016). Selenium. Adv. Nutr. 7 (2), 415–417. 10.3945/an.115.010785 26980826 PMC4785479

[B94] Qi F. Ji P. Chen Z. Wang L. Yao H. Huo M. (2021). Photosynthetic cyanobacteria-hybridized black phosphorus nanosheets for enhanced tumor photodynamic therapy. Small 17 (42), e2102113. 10.1002/smll.202102113 34524730

[B95] Qian Y. Yuan W. E. Cheng Y. Yang Y. Qu X. Fan C. (2019). Concentrically integrative bioassembly of a three-dimensional black phosphorus nanoscaffold for restoring neurogenesis, angiogenesis, and immune homeostasis. Nano Lett. 19 (12), 8990–9001. 10.1021/acs.nanolett.9b03980 31790262

[B96] Qing T. Mahmood M. Zheng Y. Biris A. S. Shi L. Casciano D. A. (2018). A genomic characterization of the influence of silver nanoparticles on bone differentiation in MC3T3-E1 cells. J. Appl. Toxicol. 38 (2), 172–179. 10.1002/jat.3528 28975650

[B97] Qing Y. Li R. Li S. Li Y. Wang X. Qin Y. (2020). Advanced black phosphorus nanomaterials for bone regeneration. Int. J. Nanomedicine 15, 2045–2058. 10.2147/IJN.S246336 32273701 PMC7104107

[B98] Qiu M. Wang D. Liang W. Liu L. Zhang Y. Chen X. (2018). Novel concept of the smart NIR-light-controlled drug release of black phosphorus nanostructure for cancer therapy. Proc. Natl. Acad. Sci. U. S. A. 115 (3), 501–506. 10.1073/pnas.1714421115 29295927 PMC5776980

[B99] Ritter J. Bielack S. S. (2010). Osteosarcoma. Ann. Oncol. 21 (7), vii320–325. 10.1093/annonc/mdq276 20943636

[B100] Rojas G. A. Hubbard A. K. Diessner B. J. Ribeiro K. B. Spector L. G. (2021). International trends in incidence of osteosarcoma (1988-2012). Int. J. Cancer 149 (5), 1044–1053. 10.1002/ijc.33673 33963769 PMC9137041

[B101] Sayed S. Faruq O. Hossain M. Im S. B. Kim Y. S. Lee B. T. (2019). Thermal cycling effect on osteogenic differentiation of MC3T3-E1 cells loaded on 3D-porous Biphasic Calcium Phosphate (BCP) scaffolds for early osteogenesis. Mater Sci. Eng. C Mater Biol. Appl. 105, 110027. 10.1016/j.msec.2019.110027 31546388

[B102] Shao J. Ruan C. Xie H. Chu P. K. Yu X. F. (2020). Photochemical activity of black phosphorus for near-infrared light controlled *in situ* biomineralization. Adv. Sci. (Weinh) 7 (14), 2000439. 10.1002/advs.202000439 32714754 PMC7375256

[B103] Shao J. Xie H. Huang H. Li Z. Sun Z. Xu Y. (2016). Biodegradable black phosphorus-based nanospheres for *in vivo* photothermal cancer therapy. Nat. Commun. 7, 12967. 10.1038/ncomms12967 27686999 PMC5056460

[B104] Shi J. Li J. Wang Y. Cheng J. Zhang C. Y. (2020). Recent advances in MoS2-based photothermal therapy for cancer and infectious disease treatment. J. Mater Chem. B 8 (27), 5793–5807. 10.1039/d0tb01018a 32597915

[B105] Shin H. Jo S. Mikos A. G. (2003). Biomimetic materials for tissue engineering. Biomaterials 24 (24), 4353–4364. 10.1016/s0142-9612(03)00339-9 12922148

[B106] Shin M. H. Park E. Y. Han S. Jung H. S. Keum D. H. Lee G. H. (2019). Multimodal cancer theranosis using hyaluronate-conjugated molybdenum disulfide. Adv. Healthc. Mater 8 (1), e1801036. 10.1002/adhm.201801036 30480380

[B107] Shui C. Scutt A. (2001). Mild heat shock induces proliferation, alkaline phosphatase activity, and mineralization in human bone marrow stromal cells and Mg-63 cells *in vitro* . J. Bone Min. Res. 16 (4), 731–741. 10.1359/jbmr.2001.16.4.731 11316001

[B108] Solak K. Mavi A. Yilmaz B. (2021). Disulfiram-loaded functionalized magnetic nanoparticles combined with copper and sodium nitroprusside in breast cancer cells. Mater Sci. Eng. C Mater Biol. Appl. 119, 111452. 10.1016/j.msec.2020.111452 33321589

[B109] Song Y. Wu H. Gao Y. Li J. Lin K. Liu B. (2020). Zinc silicate/nano-hydroxyapatite/collagen scaffolds promote angiogenesis and bone regeneration via the p38 MAPK pathway in activated monocytes. ACS Appl. Mater. Interfaces 12 (14), 16058–16075. 10.1021/acsami.0c00470 32182418

[B110] Souhami R. L. (1989). Chemotherapy for osteosarcoma. Br. J. Cancer 59 (2), 147–148. 10.1038/bjc.1989.30 2649126 PMC2247015

[B111] Stacpoole P. W. (2017). Therapeutic targeting of the pyruvate dehydrogenase complex/pyruvate dehydrogenase kinase (PDC/PDK) Axis in cancer. J. Natl. Cancer Inst. 109 (11). 10.1093/jnci/djx071 29059435

[B112] Steinbacher T. Ebnet K. (2018). The regulation of junctional actin dynamics by cell adhesion receptors. Histochem Cell Biol. 150 (4), 341–350. 10.1007/s00418-018-1691-8 29978321

[B113] Sun Z. Xie H. Tang S. Yu X. F. Guo Z. Shao J. (2015). Ultrasmall black phosphorus quantum dots: Synthesis and use as photothermal agents. Angew. Chem. Int. Ed. Engl. 54 (39), 11526–11530. 10.1002/anie.201506154 26296530

[B114] Sun Z. Zhao Y. Li Z. Cui H. Zhou Y. Li W. (2017). TiL4 -coordinated black phosphorus quantum dots as an efficient contrast agent for *in vivo* photoacoustic imaging of cancer. Small 13 (11), 1602896. 10.1002/smll.201602896 28060458

[B115] Takeuchi A. Yamamoto N. Hayashi K. Matsubara H. Miwa S. Igarashi K. (2019). Joint-preservation surgery for pediatric osteosarcoma of the knee joint. Cancer Metastasis Rev. 38 (4), 709–722. 10.1007/s10555-019-09835-z 31807972

[B116] Tan B. Wu Y. Wu Y. Shi K. Han R. Li Y. (2021). Curcumin-Microsphere/IR820 hybrid bifunctional hydrogels for *in situ* osteosarcoma chemo-co-thermal therapy and bone reconstruction. ACS Appl. Mater Interfaces 13 (27), 31542–31553. 10.1021/acsami.1c08775 34191477

[B117] Tao W. Zhu X. Yu X. Zeng X. Xiao Q. Zhang X. (2017). Black phosphorus nanosheets as a robust delivery platform for cancer theranostics. Adv. Mater 29 (1), 1603276. 10.1002/adma.201603276 PMC5205548 27797119

[B118] Tong L. Liao Q. Zhao Y. Huang H. Gao A. Zhang W. (2019). Near-infrared light control of bone regeneration with biodegradable photothermal osteoimplant. Biomaterials 193, 1–11. 10.1016/j.biomaterials.2018.12.008 30550998

[B119] Tortella G. R. Rubilar O. Duran N. Diez M. C. Martinez M. Parada J. (2020). Silver nanoparticles: Toxicity in model organisms as an overview of its hazard for human health and the environment. J. Hazard Mater 390, 121974. 10.1016/j.jhazmat.2019.121974 32062374

[B120] Truong L. B. Medina Cruz D. Mostafavi E. O'Connell C. P. Webster T. J. (2021). Advances in 3D-printed surface-modified Ca-Si bioceramic structures and their potential for bone tumor therapy. Mater. (Basel) 14 (14), 3844. 10.3390/ma14143844 PMC8306413 34300763

[B121] Tsuchiya H. Kanazawa Y. Abdel-Wanis M. E. Asada N. Abe S. Isu K. (2002). Effect of timing of pulmonary metastases identification on prognosis of patients with osteosarcoma: The Japanese musculoskeletal oncology group study. J. Clin. Oncol. 20 (16), 3470–3477. 10.1200/JCO.2002.11.028 12177108

[B122] Turnbull G. Clarke J. Picard F. Riches P. Jia L. Han F. (2018). 3D bioactive composite scaffolds for bone tissue engineering. Bioact. Mater 3 (3), 278–314. 10.1016/j.bioactmat.2017.10.001 29744467 PMC5935790

[B123] Voiry D. Fullon R. Yang J. de Carvalho Castro E. S. C. Kappera R. Bozkurt I. (2016). The role of electronic coupling between substrate and 2D MoS2 nanosheets in electrocatalytic production of hydrogen. Nat. Mater 15 (9), 1003–1009. 10.1038/nmat4660 27295098

[B124] Wang J L J. L. Xu J. K. Hopkins C. Chow D. H. Qin L. (2020). Biodegradable magnesium-based implants in orthopedics-A general review and perspectives. Adv. Sci. (Weinh) 7 (8), 1902443. 10.1002/advs.201902443 32328412 PMC7175270

[B125] Wang L. Hu P. Jiang H. Zhao J. Tang J. Jiang D. (2022). Mild hyperthermia-mediated osteogenesis and angiogenesis play a critical role in magnetothermal composite-induced bone regeneration. Nano Today 43, 101401. 10.1016/j.nantod.2022.101401

[B126] Wang R. Liu W. Wang Q. Li G. Wan B. Sun Y. (2020). Anti-osteosarcoma effect of hydroxyapatite nanoparticles both *in vitro* and *in vivo* by downregulating the FAK/PI3K/Akt signaling pathway. Biomater. Sci. 8 (16), 4426–4437. 10.1039/d0bm00898b 32618992

[B127] Wang S. Shao J. Li Z. Ren Q. Yu X. F. Liu S. (2019). Black phosphorus-based multimodal nanoagent: Showing targeted combinatory therapeutics against cancer metastasis. Nano Lett. 19 (8), 5587–5594. 10.1021/acs.nanolett.9b02127 31260628

[B128] Wang Y. Hu X. Zhang L. Zhu C. Wang J. Li Y. (2019). Bioinspired extracellular vesicles embedded with black phosphorus for molecular recognition-guided biomineralization. Nat. Commun. 10 (1), 2829. 10.1038/s41467-019-10761-5 31249296 PMC6597708

[B129] Wang Z. Tang X. Wang X. Yang D. Yang C. Lou Y. (2016). Near-infrared light-induced dissociation of zeolitic imidazole framework-8 (ZIF-8) with encapsulated CuS nanoparticles and their application as a therapeutic nanoplatform. Chem. Commun. (Camb) 52 (82), 12210–12213. 10.1039/c6cc06616j 27722473

[B130] Wang Z. X. Chen C. Y. Wang Y. Li F. X. Z. Huang J. Luo Z. W. (2019). Ångstrom‐scale silver particles as a promising agent for low‐toxicity broad‐spectrum potent anticancer therapy. Adv. Funct. Mater. 29 (23), 1808556. 10.1002/adfm.201808556

[B131] Wang Z. Zhao J. Tang W. Hu L. Chen X. Su Y. (2019). Multifunctional nanoengineered hydrogels consisting of black phosphorus nanosheets upregulate bone formation. Small 15 (41), e1901560. 10.1002/smll.201901560 31423735

[B132] Ward W. G. Mikaelian K. Dorey F. Mirra J. M. Sassoon A. Holmes E. C. (1994). Pulmonary metastases of stage IIB extremity osteosarcoma and subsequent pulmonary metastases. J. Clin. Oncol. 12 (9), 1849–1858. 10.1200/JCO.1994.12.9.1849 8083708

[B133] Windhagen H. Radtke K. Weizbauer A. Diekmann J. Noll Y. Kreimeyer U. (2013). Biodegradable magnesium-based screw clinically equivalent to titanium screw in hallux valgus surgery: Short term results of the first prospective, randomized, controlled clinical pilot study. Biomed. Eng. Online 12, 62. 10.1186/1475-925X-12-62 23819489 PMC3702514

[B134] Wong K. C. Kumta S. M. (2013). Joint-preserving tumor resection and reconstruction using image-guided computer navigation. Clin. Orthop. Relat. Res. 471 (3), 762–773. 10.1007/s11999-012-2536-8 22948524 PMC3563823

[B135] Woolbright B. L. Rajendran G. Harris R. A. Taylor J. A. 3rd (2019). Metabolic flexibility in cancer: Targeting the pyruvate dehydrogenase kinase:pyruvate dehydrogenase Axis. Mol. Cancer Ther. 18 (10), 1673–1681. 10.1158/1535-7163.MCT-19-0079 31511353

[B136] Wu J. (2021). The enhanced permeability and retention (EPR) effect: The significance of the concept and methods to enhance its application. J. Pers. Med. 11 (8), 771. 10.3390/jpm11080771 34442415 PMC8402171

[B137] Wu Y. He G. Zhang Y. Liu Y. Li M. Wang X. (2016). Unique antitumor property of the Mg-Ca-Sr alloys with addition of Zn. Sci. Rep. 6, 21736. 10.1038/srep21736 26907515 PMC4764862

[B138] Xie Z. Peng M. Lu R. Meng X. Liang W. Li Z. (2020). Black phosphorus-based photothermal therapy with aCD47-mediated immune checkpoint blockade for enhanced cancer immunotherapy. Light Sci. Appl. 9, 161. 10.1038/s41377-020-00388-3 33014356 PMC7492464

[B139] Xu L. Wang Y. Y. Huang J. Chen C. Y. Wang Z. X. Xie H. (2020). Silver nanoparticles: Synthesis, medical applications and biosafety. Theranostics 10 (20), 8996–9031. 10.7150/thno.45413 32802176 PMC7415816

[B140] Xu Y. Ren F. Liu H. Zhang H. Han Y. Liu Z. (2019). Cholesterol-modified black phosphorus nanospheres for the first NIR-II fluorescence bioimaging. ACS Appl. Mater Interfaces 11 (24), 21399–21407. 10.1021/acsami.9b05825 31120234

[B141] Yang B. Yin J. Chen Y. Pan S. Yao H. Gao Y. (2018). 2D-Black-Phosphorus-Reinforced 3D-printed scaffolds:A stepwise countermeasure for osteosarcoma. Adv. Mater 30 (10), 1705611. 10.1002/adma.201705611 29333689

[B142] Yang D. Yang G. Yang P. Lv R. Gai S. Li C. (2017). Assembly of Au plasmonic photothermal agent and iron oxide nanoparticles on ultrathin black phosphorus for targeted photothermal and photodynamic cancer therapy. Adv. Funct. Mater. 27 (18), 1700371. 10.1002/adfm.201700371

[B143] Yang J. Qin H. Chai Y. Zhang P. Chen Y. Yang K. (2021). Molecular mechanisms of osteogenesis and antibacterial activity of Cu-bearing Ti alloy in a bone defect model with infection *in vivo* . J. Orthop. Transl. 27, 77–89. 10.1016/j.jot.2020.10.004 PMC7779545 33437640

[B144] Yang N. Gong F. Cheng L. Lei H. Li W. Sun Z. (2021). Biodegradable magnesium alloy with eddy thermal effect for effective and accurate magnetic hyperthermia ablation of tumors. Natl. Sci. Rev. 8 (1), nwaa122. 10.1093/nsr/nwaa122 34691551 PMC8288380

[B145] Yang Q. Yin H. Xu T. Zhu D. Yin J. Chen Y. (2020). Engineering 2D mesoporous Silica@MXene-integrated 3D-printing scaffolds for combinatory osteosarcoma therapy and NO-augmented bone regeneration. Small 16 (14), e1906814. 10.1002/smll.201906814 32108432

[B146] Yang Y. Guo L. Wang Z. Liu P. Liu X. Ding J. (2021c). Targeted silver nanoparticles for rheumatoid arthritis therapy via macrophage apoptosis and Re-polarization. Biomaterials 264, 120390. 10.1016/j.biomaterials.2020.120390 32980634

[B147] Yang Y. Tian Q. Wu S. Li Y. Yang K. Yan Y. (2021d). Blue light-triggered Fe(2+)-release from monodispersed ferrihydrite nanoparticles for cancer iron therapy. Biomaterials 271, 120739. 10.1016/j.biomaterials.2021.120739 33690102

[B148] Yin F. Hu K. Chen S. Wang D. Zhang J. Xie M. (2017). Black phosphorus quantum dot based novel siRNA delivery systems in human pluripotent teratoma PA-1 cells. J. Mater Chem. B 5 (27), 5433–5440. 10.1039/c7tb01068k 32264082

[B149] Zafeiris K. Brasinika D. Karatza A. Koumoulos E. Karoussis I. K. Kyriakidou K. (2021). Additive manufacturing of hydroxyapatite-chitosan-genipin composite scaffolds for bone tissue engineering applications. Mater Sci. Eng. C Mater Biol. Appl. 119, 111639. 10.1016/j.msec.2020.111639 33321677

[B150] Zakhireh S. Adibkia K. Beygi-Khosrowshahi Y. Barzegar-Jalali M. (2021). Osteogenesis promotion of selenium-doped hydroxyapatite for application as bone scaffold. Biol. Trace Elem. Res. 199 (5), 1802–1811. 10.1007/s12011-020-02309-2 32816138

[B151] Zan R. Ji W. Qiao S. Wu H. Wang W. Ji T. (2020). Biodegradable magnesium implants: A potential scaffold for bone tumor patients. Sci. China Mater. 64 (4), 1007–1020. 10.1007/s40843-020-1509-2

[B152] Zan R. Wang H. Cai W. Ni J. Luthringer-Feyerabend B. J. C. Wang W. (2022). Controlled release of hydrogen by implantation of magnesium induces P53-mediated tumor cells apoptosis. Bioact. Mater 9, 385–396. 10.1016/j.bioactmat.2021.07.026 34820578 PMC8586587

[B153] Zeng H. Cao J. J. Combs G. F. Jr. (2013). Selenium in bone health: Roles in antioxidant protection and cell proliferation. Nutrients 5 (1), 97–110. 10.3390/nu5010097 23306191 PMC3571640

[B154] Zeng X. Luo M. Liu G. Wang X. Tao W. Lin Y. (2018). Polydopamine-modified black phosphorous nanocapsule with enhanced stability and photothermal performance for tumor multimodal treatments. Adv. Sci. (Weinh) 5 (10), 1800510. 10.1002/advs.201800510 30356942 PMC6193171

[B155] Zhang R. Lee P. Lui V. C. Chen Y. Liu X. Lok C. N. (2015). Silver nanoparticles promote osteogenesis of mesenchymal stem cells and improve bone fracture healing in osteogenesis mechanism mouse model. Nanomedicine 11 (8), 1949–1959. 10.1016/j.nano.2015.07.016 26282383

[B156] Zhang W. Yu L. Jiang Y. Guo C. (2021). Phycocyanin-functionalized black phosphorus quantum dots enhance PDT/PTT therapy by inducing ROS and irreparable DNA damage. Biomater. Sci. 9 (15), 5302–5318. 10.1039/d1bm00106j 34184011

[B157] Zhang X. F. Liu Z. G. Shen W. Gurunathan S. (2016). Silver nanoparticles: Synthesis, characterization, properties, applications, and therapeutic approaches. Int. J. Mol. Sci. 17 (9), 1534. 10.3390/ijms17091534 27649147 PMC5037809

[B158] Zhang X. Li X. Zheng C. Yang C. Zhang R. Wang A. (2022). Ferroptosis, a new form of cell death defined after radiation exposure. Int. J. Radiat. Biol. 98, 1201–1209. 10.1080/09553002.2022.2020358 34982648

[B159] Zhang X. Xie H. Liu Z. Tan C. Luo Z. Li H. (2015). Black phosphorus quantum dots. Angew. Chem. Int. Ed. Engl. 54 (12), 3653–3657. 10.1002/anie.201409400 25649505

[B160] Zhang Y. Xu J. Ruan Y. C. Yu M. K. O'Laughlin M. Wise H. (2016). Implant-derived magnesium induces local neuronal production of CGRP to improve bone-fracture healing in rats. Nat. Med. 22 (10), 1160–1169. 10.1038/nm.4162 27571347 PMC5293535

[B161] Zhao D. Huang S. Lu F. Wang B. Yang L. Qin L. (2016). Vascularized bone grafting fixed by biodegradable magnesium screw for treating osteonecrosis of the femoral head. Biomaterials 81, 84–92. 10.1016/j.biomaterials.2015.11.038 26724456

[B162] Zhao D. Witte F. Lu F. Wang J. Li J. Qin L. (2017). Current status on clinical applications of magnesium-based orthopaedic implants: A review from clinical translational perspective. Biomaterials 112, 287–302. 10.1016/j.biomaterials.2016.10.017 27770632

[B163] Zhao Y. Peng X. Xu X. Wu M. Sun F. Xin Q. (2023). Chitosan based photothermal scaffold fighting against bone tumor-related complications: Recurrence, infection, and defects. Carbohydr. Polym. 300, 120264. 10.1016/j.carbpol.2022.120264 36372515

[B164] Zhou W. Zhang Y. Meng S. Xing C. Ma M. Liu Z. (2021). Micro-/Nano-Structures on biodegradable magnesium@PLGA and their cytotoxicity, photothermal, and anti-tumor effects. Small Methods 5 (2), e2000920. 10.1002/smtd.202000920 34927892

[B165] Zhu X. Li L. Tang J. Yang C. Yu H. Liu K. (2022). Cascade-responsive nano-assembly for efficient photothermal-chemo synergistic inhibition of tumor metastasis by targeting cancer stem cells. Biomaterials 280, 121305. 10.1016/j.biomaterials.2021.121305 34890970

