# Supplementary figures and images for: Multifunctional inorganic biomaterials: New weapons targeting osteosarcoma
Source: Front Mol Biosci. 2023 Jan 4;9:1105540. doi: 10.3389/fmolb.2022.1105540 (PMC9846365; doi:10.3389/fmolb.2022.1105540)

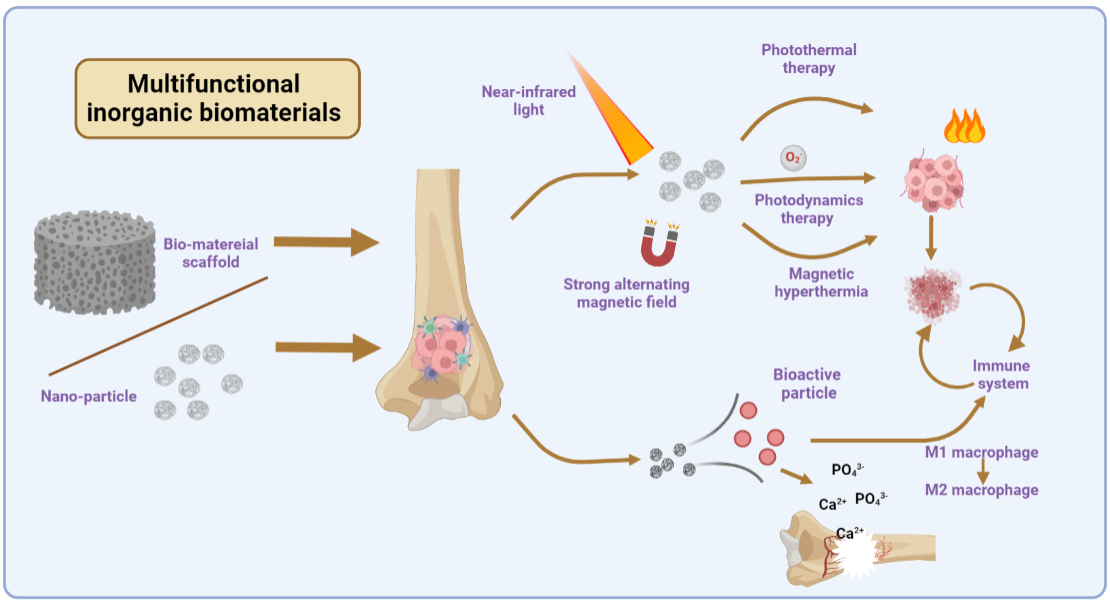

Supplement: Supplementary file 1 [file Table2.DOCX]
